# Antiproliferative Water-Soluble Mono- and Binuclear Ruthenium Complexes with Pyridone–Imidazole Ligands

**DOI:** 10.3390/ijms26115214

**Published:** 2025-05-29

**Authors:** Ilya A. Shutkov, Nikolai A. Melnichuk, Sofya A. Ovakimyan, Dmitrii M. Mazur, Nataliya E. Borisova, Maxim L. Kuznetsov, Ivan A. Godovikov, Konstantin A. Lyssenko, Dmitrii S. Yakovlev, Alexander A. Spasov, Elena R. Milaeva, Alexey A. Nazarov

**Affiliations:** 1 Department of Chemistry, M.V. Lomonosov Moscow State University, Leninskie Gory 1/3, 119991 Moscow, Russiamilaeva@med.chem.msu.ru (E.R.M.); 2 Centro de Química Estrutural, Institute of Molecular Sciences, Departamento de Engenharia Química, Instituto Superior Técnico, Universidade de Lisboa, 1049-001 Lisboa, Portugal; 3 A.N. Nesmeyanov Institute of Organoelement Compounds of Russian Academy of Sciences (INEOS RAS), Vavilov Street 28, Building 1, 119991 Moscow, Russia; 4 Scientific Center for Innovative Drugs, Volgograd State Medical University, Novorossiyskaya Street 39, 400087 Volgograd, Russia

**Keywords:** ruthenium, metallacycles, antitumor compounds, antiproliferative activity, cytotoxicity, maltol, pyridones, water solubility

## Abstract

In this study, we synthesized and characterized new imidazole ligands containing pyridone groups, as well as mononuclear and binuclear ruthenium complexes, which are a new class of water-soluble metallacycles. We studied the antiproliferative activity of these compounds in vitro using the MTT assay on a panel of human cancer cell lines and on primary rat fibroblasts, where we observed a complete absence of cytotoxicity up to a concentration of 1000 µM. For the binuclear metallocycle compounds, we investigated their solubility in water, resistance to hydrolysis, and ability to induce apoptosis in tumor cells.

## 1. Introduction

**Figure 1:**
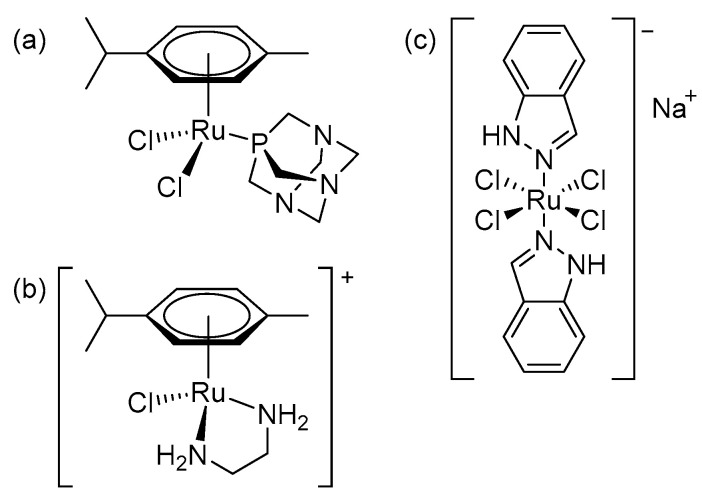
Structures of ruthenium lead compounds: RAPTA-C (**a**), RAED-C (**b**), BOLD-100 (**c**).

In the search for a potential replacement for platinum drugs for cancer chemotherapy, ruthenium compounds seem to be the most promising so far. Thus, in 2021, the Ru(III) coordination compound BOLD-100 (Figure 1c) was approved by the FDA as an orphan drug for the treatment of stomach cancer [1]. Also, the development and testing of Ru(II) organometallic compounds such as RAPTA and RAED [2,3,4,5,6,7], as well as their analogues [8,9,10], are still actively underway (Figure 1a,b).

**Figure 2:**
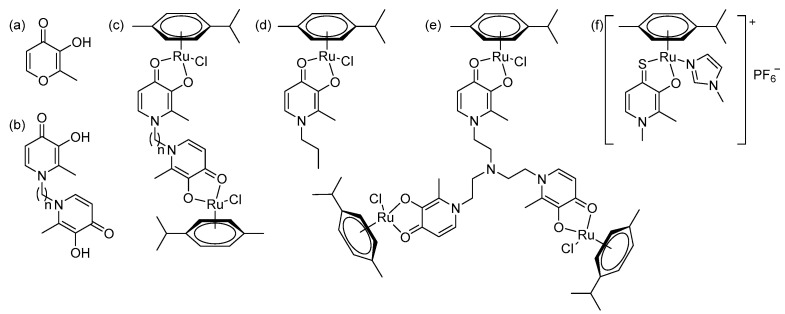
Structures of maltol (**a**), pyridone-derived ligands (**b**), bi- (**c**), mono- (**d**), and trinuclear (**e**) ruthenium complexes with pyridone ligands, and thiopyridone ruthenium complex-carrying 1-methylimidazole (**f**).

Important pharmacokinetic parameters of potential antitumor ruthenium compounds include stability in physiological media, water solubility, and lipophilicity. These parameters directly affect the antitumor activity of the compounds. The environment of the ruthenium atom greatly influences these parameters, and the choice of ligands used in the compounds is therefore crucial. Organic ruthenium complexes are susceptible to ligand exchange reactions. In aqueous media, these organometallic compounds with an arene ligand undergo hydrolysis to form ruthenium dimers with hydroxide bridges. Due to their high stability, these dimers cannot bind to biological targets [11,12,13,14,15]. For example, O,O-chelate bidentate ligands—pyrones, such as maltol (Figure 2a)—have proven themselves well. The interest in ruthenium complexes with pyrone ligands arises from the increased stability and water solubility of ruthenium compounds, which are achieved through the incorporation of a pyrone structure into the molecular framework.

In the search for promising antitumor agents of ruthenium, many O,O-chelate complexes based of maltol, other pyrones and thio-/selenium derivatives have been obtained and tested. Most of the known complexes have shown high antiproliferative activity [16,17,18]. A new possible molecular target was proposed for such complexes: topoisomerase IIα, which is promising in the design of new antitumor agents. Topoisomerase IIα (topo2α) is the basis of a nuclear enzyme, is involved in DNA replication and transcription, and is significantly expressed in many tumors [19,20,21,22]. In addition, mono-, bi-, and trinuclear complexes were obtained and described, their antitumor activity was compared, and binuclear complexes turned out to be the most promising (Figure 2c–e). However, the ruthenium complexes with pyrone ligands, which were obtained in several studies, were found to be unstable. This is probably due to the stabilization of the water coordination with ruthenium through the O,O-chelate system. The authors proposed a way to increase the stability of these complexes in ligand exchange reactions by replacing the labile halide ligands with a heterocyclic compound, such as 1-methylimidazole (Figure 2f). More stable analogues showed higher antitumor activity and the ability to inhibit topoisomerase in in vitro tests [23,24].

In this work, we propose a novel approach to the synthesis of imidazole-modified pyridone ligands. These ligands can be coordinated to a ruthenium atom through both the imidazole and pyridone fragments, due to the formation of an O,O-chelated structure. The proposed ligands were employed in the synthesis of novel Ru(III) coordination complexes and mononuclear organometallic Ru(II) compounds. Additionally, a new class of cyclic binuclear ruthenium complexes were obtained, where each ruthenium atom was bonded to both a pyridone and imidazole fragment. This allowed us to address the challenge of stability in ligand exchange reactions during molecular assembly.

## 2. Results and Discussion

### 2.1. Synthesis and Characterization

**Figure 3:**
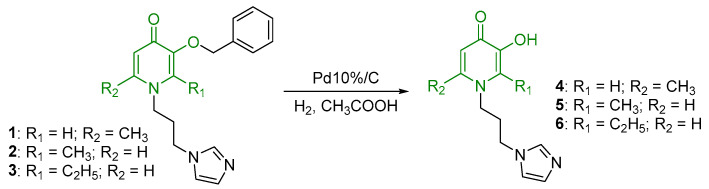
Removal of benzyl protective group through hydrogenolysis.

Imidazole-based ligands with pyridone fragments **1**–**3** were obtained using a procedure in the literature, starting from benzyloxy derivatives of maltol, ethylmaltol, and allomaltol [8]. In this work, in order to obtain ligands **4**–**6** with an unsubstituted maltol fragment, allomaltol, and ethylmaltol, as well as to further synthesize O,O-chelated complexes with these ligands, the benzyl group was removed through hydrogenolysis. (Figure 3). Hydrogenolysis catalyzed by 10% palladium on coal was carried out in acetic acid for 10 h and the products **4**–**6** were isolated by column chromatography on silica gel (eluent: methanol). Hygroscopic beige powders were obtained. The composition and structure of these powders were confirmed using X-ray diffraction analysis, nuclear magnetic resonance (NMR) spectroscopy, electrospray ionization mass spectrometry (ESI MS), and elemental analysis. The purity of the powders was also confirmed by elemental analysis. NMR signals were assigned using ^1^H^1^H COSY, ^1^H^13^C HSQC, ^1^H^13^C HMBC correlation NMR spectroscopy. No signals from the benzyl group protons were observed in the NMR spectra of compounds **4**–**6**, indicating the successful removal of the protective group.

X-ray diffraction investigation of the **4**–**6** ligands reveals that **5** and **6** crystallize with two or a half water molecule, while only ligand **4** crystallizes without solvate molecules in the unit cell. The principal bond lengths and angles in the ligands are almost the same. In particular, the C-OH and C=C bonds are equal to 1.35 and 1.26 Å. It should be noted that these bond length values are within 0.02 Å and are observed in corresponding complexes. In a crystal, four molecules are assembled into the planar 10-membered H-bonded cycle by means of two O-H…O bonds (O…O 2.594(1) Å).

In the crystal of **6**, a water molecule occupying the special position axis two forms N-H…O H-bonds with imidazole (N…O 2.919(2) Å). In this case, the imidazole cycles of neighbouring molecules are tightened by hydrogen bonds into a stacking dimer with the shortest C…C distance of 3.325 Å. It should be noted that in the other structures, the stacking interaction is not realised, which suggests that the interaction of the pi systems is forced in nature. The above mentioned 10-membered ring (O…O 2.635(1) Å) is also observed in **6**, but it is significantly folded. Finally, in **5**, the presence of two solvate water molecules excludes direct supramolecular interaction of the molecules in the crystal. In the crystal, we can identify six-membered cycles including four water molecules and two imidazole nitrogen atoms located in para-positions. OH and C=O are also bonded to the water atoms of these cycles.

**Figure 4:**
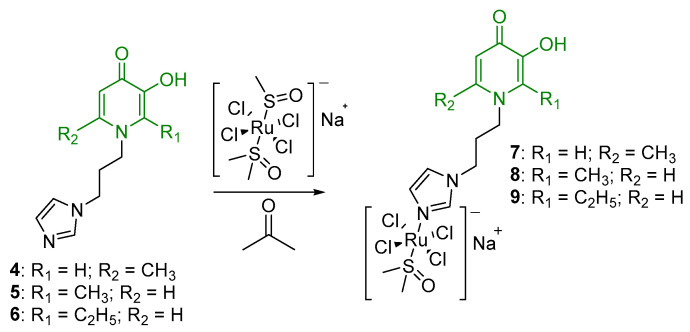
Synthesis of Ru(III) complexes with pyridone moiety.

Ru(III) complexes with imidazole-modified pyridone ligands were obtained by replacing the DMSO molecule in the initial sodium salt of the Ru(III) compound with the imidazole ligand (Figure 4). The reaction was carried out in acetone for 12 h. The desired complexes were isolated using column flash chromatography on silica gel, using acetone as the eluent. However, complexes **7**–**9** proved to be hydrolytically unstable, even in powder form, likely due to the hygroscopic nature of the ligands. As a result, they could not be purified and isolated in their pure form.

**Figure 5:**
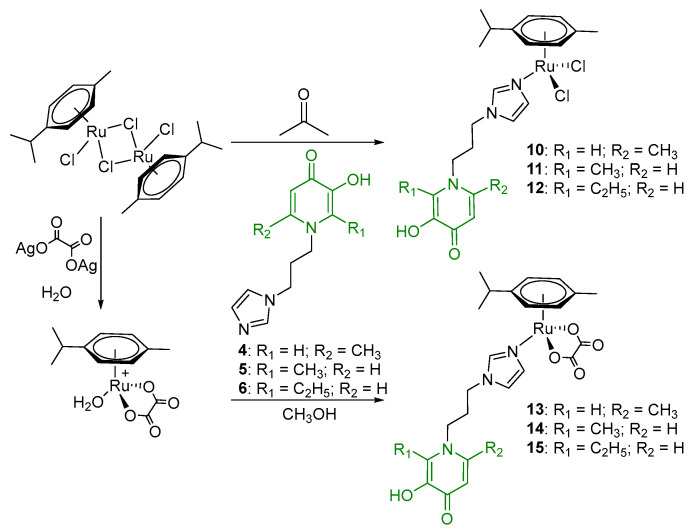
Synthesis of Ru(II) mononuclear complexes.

The mononuclear organometallic Ru(II) compounds with imidazole-modified pyridone and chloride ligands **10**–**12** were obtained by interaction of a dimeric ruthenium complex [(η^6^*-p*-cymene)RuCl_2_]_2_ with ligands **4**–**6** (Figure 5). These complexes were also found to be unstable, likely due to hydrolysis of the Ru-Cl bonds, and were not isolated in their pure form.

Using the previously described method [8,9] of increasing the stability of organometallic compounds in ligand exchange reactions by replacing chloride ligands with a dicarboxylic acid group, analogues with an oxalate moiety were synthesized in this study. At the first stage, a complex with an oxalate group was formed in the reaction between the dimer [(η^6^*-p*-cymene)RuCl_2_]_2_ and silver oxalate in water. This complex was then reacted with ligands **4**–**6** in methanol (Figure 5).

The reaction product was isolated using column chromatography on silica gel with methanol as the eluent. The composition and structure of compounds **13**–**15** were confirmed using NMR spectroscopy, ESI MS, and elemental analysis to determine purity. NMR signals were assigned using ^1^H^1^H COSY, ^1^H^13^C HSQC, ^1^H^13^C HMBC correlation NMR spectroscopy.

**Figure 6:**
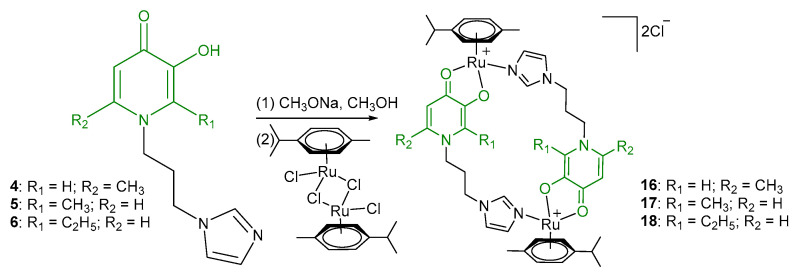
Synthesis of Ru(II) binuclear metallacycles.

In the ^1^H NMR spectra of oxalate complexes **13**–**15**, characteristic shifts could be identified that indicate the formation of these complexes. The shifts in the signals of aromatic and imidazole protons relative to those of the ligands were observed. Upon coordination of ligands **4**–**6**, there were changes in the proton resonances of both the imidazole ring and the pyridone fragment, as well as the appearance of new signals from the protons of the aromatic fragment of the ruthenium center. It is known that maltol and its analogues tend to form O,O-chelate complexes, which have antitumor activity [16,17,18]. Ligands **4**–**6** containing pyridone and imidazole fragments were capable of coordination to the ruthenium atom by both the imidazole nitrogen atom and the oxygen atoms of the pyridone fragment. A necessary condition for obtaining O,O-chelate complexes is preliminary deprotonation. The complexes were obtained in two stages (Figure 6). At the first stage, ligands **4**–**6** were deprotonated with sodium methoxide in methanol and were then reacted with the [(η^6^*-p*-cymene)RuCl_2_]_2_ dimer. The reaction mixture was evaporated, the by-products were precipitated with diethyl ether and separated, and then the mother liquor was evaporated. Pure compounds **16**–**18** were isolated by crystallization from a mixture of methanol and diethyl ether, and red-orange crystalline substances were obtained. The composition and structure of complexes **16**–**18** were confirmed by X-ray diffraction analysis, NMR spectroscopy, and ESI MS, and the purity was determined by elemental analysis.

**Figure 7:**
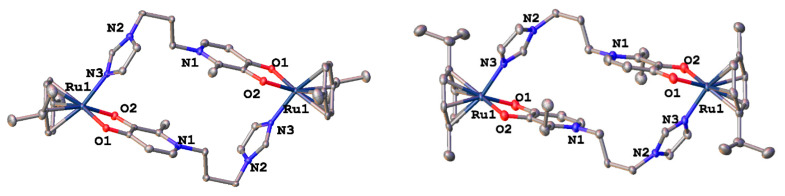
Molecular structure of complex **17** (**left**) and **18** (**right**) in representation of atoms by atomic displacement parameters (*p* = 50%).

X-ray diffraction analysis of crystals of complexes **16**–**18** showed that the complexes are cationic cyclic structures with two ruthenium centers and two imidazole-pyridone ligands between them (Figure 7). Each of the ruthenium atoms is coordinated both by the N atom of imidazole and by the O atoms of the maltol fragment or its analogues (ethyl maltol and allomaltol). The mutual arrangement of the RuO_3_N fragment and the benzene ring is such that the Ru-O and Ru-N bonds are located above the middle of C-C bonds of the coordinated aryl, which leads to an alternation of bonds in the ring with their elongation for the bonds located below the Ru-O(Ru-N) bonds, which vary between 1.425(5)–1.438(5) Å compared to the rest in the ring (1.399(5)–1.402(5) Å).

According to X-ray diffraction data, in crystals of complexes **17** and **18**, dications are located at the center of symmetry. It should be noted that although the pyridone cycles are parallel to each other, the absence of overlapping of π systems, as well as the distance between the planes equal to ca. 4, unambiguously indicates the absence of any stacking interaction Å.

Complex **17** crystallizes in the form of solvates with four molecules of methanol and two molecules of water, and complex **18** with four molecules of methanol, while the solvate molecules form associates with chloride anions. The main crystallographic constants and refinement parameters are presented in Table S1.

**Figure 8:**
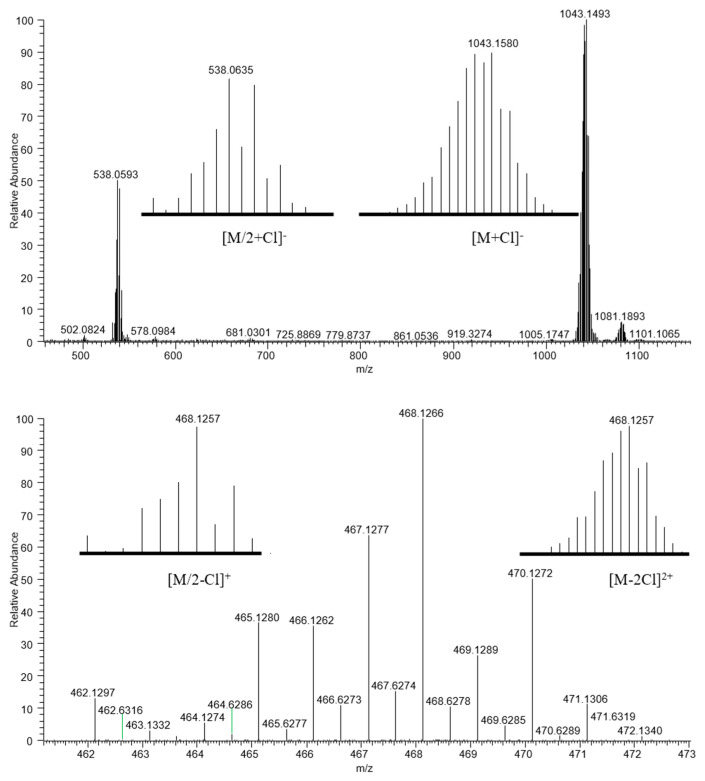
ESI mass spectra of compound **17** (negative ion mode (**top**) and positive ion mode (**bottom**)).

ESI mass spectra of compounds **16**–**18** in the negative ion mode revealed two signals of adducts with the chloride ion showing *m*/*z* values corresponding to the [M + Cl]^−^ anion and half of the target dimer [(η^6^-p-cymene)Ru(L(**16**–**18**))Cl]^−^. Both demonstrated clear and characteristic isotopic distribution of ruthenium, proving the proposed formula. Positive ion ESI mass spectra contained overlapped signals with the same *m*/*z* value, which can be attributed to the double-charged cation [M − 2Cl]^2+^ and the single-charged cation [M/2 − Cl]^+^ (Figure 8). The observed signals fully correspond to the calculated ones in terms of *m*/*z* values and isotopic distribution.

**Figure 9:**
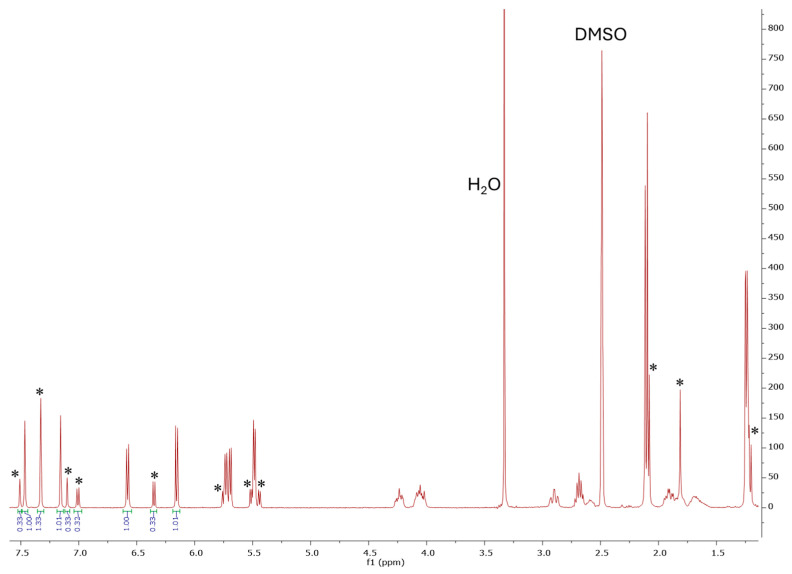
^1^H NMR spectrum of binuclear metallacycle **17** (* minor set of signals).

In the ^1^H NMR spectra of complexes **16**–**18**, a doubling of the signals of all types of protons with different integral intensities included in the compounds were observed (Figure 9), which can be explained by the conformational mobility of the macrocycle. In this case, relative to corresponding ligands **4**–**6**, a shift in the signals of the protons of the imidazole and pyridone fragments was observed, as well as the appearance of additional signals from aromatic protons of the ruthenium center.

**Figure 10:**
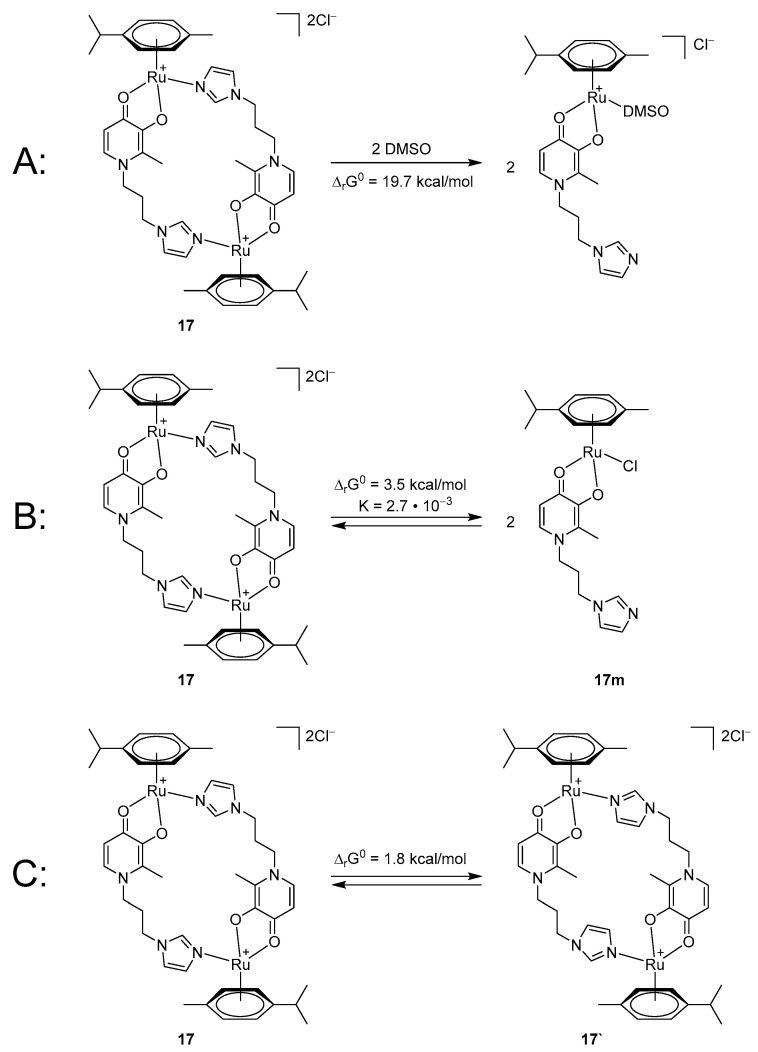
Calculated transformations of **17**.

It is known that DMSO molecules can coordinate a metal atom and produce products of ligand exchange reactions. In order to exclude the formation of ligand exchange products, which may correspond to the second set of signals, the NMR spectrum in acetone was recorded, in which a secondary set of signals of all protons was also observed. Moreover, the integral ratio of the main set of signals to the secondary one depended on the nature of the pyridone fragment of the ligand. The DFT calculations also did not support this hypothesis. The Gibbs free energy of the dimer **17** decomposition followed by the DMSO coordination (Figure 10A) was found to be 19.7 kcal/mol.

As a result, we put forward two hypotheses, i.e., the monomer–dimer equilibrium and the formation of two isomers (enantiomers or conformers). The calculated Gibbs free energy of the dimer **17** decomposition into two monomers **17m** (Figure 10B) is 3.5 kcal/mol, which corresponds to the equilibrium constant of 2.7 × 10^–3^. Assuming the initial concentration of **17** to be 0.1 M, this equilibrium constant corresponds to the equilibrium dimer:monomer ratio of 5.8:1 that is in a qualitative agreement with the experimental ^1^H NMR integral ratio of 3.1:1.

The optimized structure of another conformer **17’** obtained from **17** by rotation of two imidazole rings was also calculated (Figure 10C). Isomer **17** (from the X-ray structure) was found to be more stable than **17’** by 1.8 kcal/mol, which corresponds to the **17**:**17’** isomeric ratio of 21:1. Thus, the DFT calculations support the first hypothesis.

To unambiguously correlate the signals of protons and carbons in the structure of binuclear metallacycles **16**–**18**, as well as in an attempt to explain the presence of a second set of signals, 2D spectra were recorded using NMR correlation methods: COSY, HSQC and HMBC. Interestingly, the protons of the alkyl linker were not equivalent in the main set of signals, and each proton of the CH_2_ groups was represented in the spectrum by a separate signal, which can be explained by the conformational fixation of the mobile alkyl linker in the structure of the metallocycle.

**Figure 11:**
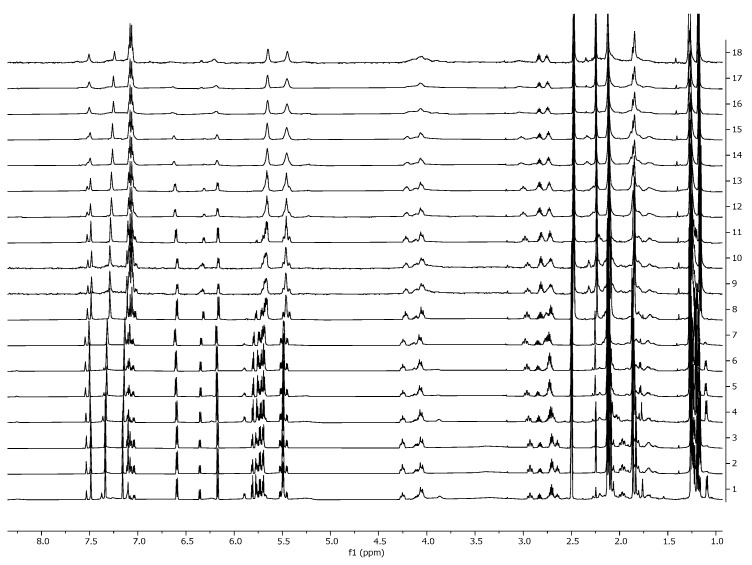
A series of ^1^H NMR spectra recorded at different temperatures (ΔT = +5 K from bottom to top).

For compound **17**, NOESY or ROESY spectra were additionally recorded, and a temperature-dependent NMR experiment was performed (Figure 11). It is worth noting that after cooling, the spectrum returned to its original form, which indicates that no irreversible processes in DMSO occur when the temperature of the solution increases.

**Figure 12:**
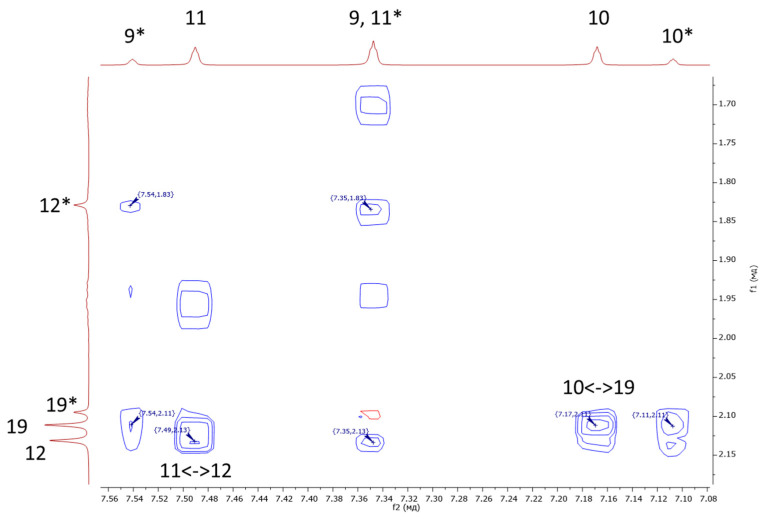
^1^H^1^H-ROESY spectrum of complex **17** color indicated intensity of peaks.

**Figure 13:**
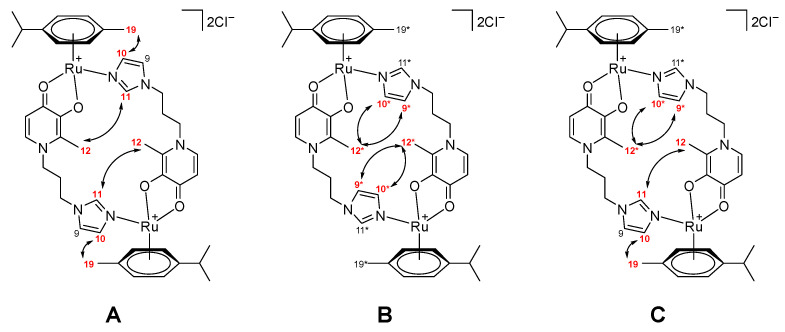
Structures of possible conformers (**A**–**C**) of complex **17** in solution.

For complex **17**, we performed a more detailed analysis using NMR spectroscopy to determine the relative positions of the diazole ring substituents. We obtained the ^1^H-^1^H-ROESY spectrum shown in Figure 12. From the figure, it can be seen that there are two intense two-dimensional signals corresponding to pairwise interactions between the positions 11 and 12 (methyl substituents) and 10 and 19 (methyl substituents). These interactions are due to the spatial convergence of these positions in one conformation (Figure 13, conformer A). Additionally, there is a weaker signal corresponding to an interaction between positions 9* and 12* (methyl substituents of the second conformer), due to the spatial proximity of these positions in the second possible conformation (Figure 13, conformer B). All this indicates that at least two dimeric conformers of complex **17** may be present in the solution in a ratio of 1:3; the conformer structures are shown in Figure 13.

It is also possible that, in addition to the “pure” conformers **A** and **B**, a mixed conformation **C** (Figure 13, conformer **C**) of compound **17** is present in the solution. However, the overall ratio of the mutual “orientations” of the discussed fragments in all three possible conformations, **A**–**C**, is approximately 1:3.

**Figure 14:**
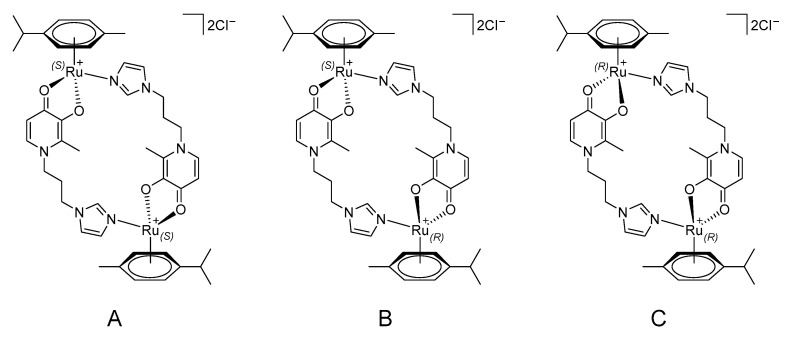
Structures of possible isomers (**A**–**C**) of complex **17** in solution.

However, the ^1^H^1^H-ROESY spectrum also contains signals corresponding to the interaction between positions 11* and 12*, as well as 10* and 19*, and the proton signal at position 12* is shifted by almost 0.3 ppm, which may indicate the presence of several isomers in the solution (Figure 14). Moreover, the phenomenon of optical activity of ruthenium was observed in other work for structurally similar complexes [25,26].

Attempts were made to separate the products using chromatography on silica gel, but it was not possible to isolate the substances due to the irreversible adsorption of the complexes onto the silica gel.

One of the most important pharmacokinetic parameters of potential drug candidates is solubility in water and stability in aqueous solutions. The water solubility of metallocycles was evaluated using spectrophotometry. For this purpose, a saturated solution of the compound was obtained and diluted several tens of times. The concentration of the resulting solution was determined according to a calibration graph of the dependence of the optical density of the absorption maximum (λ = 332 nm) on the concentration. The solubility data is shown in Table 1.

The resistance of binuclear complexes to hydrolysis was studied by spectrophotometry. Solutions of the studied complex were prepared in dry DMF and in a mixture of DMF and water (9:1) with the same concentration, and absorption spectra were recorded. No changes were observed in the spectrum of the compound over 24 h, which indicates high resistance of the binuclear complexes to hydrolysis.

### 2.2. Antiproliferative Activity Study

To assess antiproliferative activity, the MTT assay was used, which is widely used in the study of in vitro cytotoxicity. Studies of the antiproliferative activity of all obtained compounds were carried out on human cancer cell lines: A549 (lung carcinoma), HCT116 (colon carcinoma), MCF7 (breast adenocarcinoma), and SW480 (colon adenocarcinoma). The data obtained are presented in Table 2. All measurements were carried out in three independent replicates in comparison with the clinically used anticancer drug cisplatin as an internal standard.

Comparison with the activity of previously synthesized complexes with a benzyl protecting group in the pyridone fragment [8] showed that the removal of this group led to moderate antiproliferative activity for ligands **5** and **6** and for complexes **14** and **15** containing free maltol or ethylmaltol. Additionally, as has been previously shown for Ru(II)-based organometallic complexes with other imidazole ligands [9], replacing chloride ligands with oxalate fragments in order to obtain stable complexes that do not undergo ligand exchange reactions does not result in a loss of antitumor activity. Macrocyclic binuclear complexes **17** and **18** exhibited a significant increase in activity at low micromolar concentrations compared to their parent ligands and mononuclear counterparts. In all cases, the more lipophilic compounds based on ethyl maltol were more active. It is noteworthy that allomaltol-based compounds **4**, **13**, and **16** were found to be low in toxicity, which cannot be solely explained by a change in the position of the methyl group and warrants further investigation. To investigate the mechanism of tumor cell death based on the analysis of the obtained data on antiproliferative activity in vitro, binuclear complexes **17** and **18,** with a fragment of maltol and ethylmaltol, were selected as leader compounds for new complexes.

**Figure 15:**
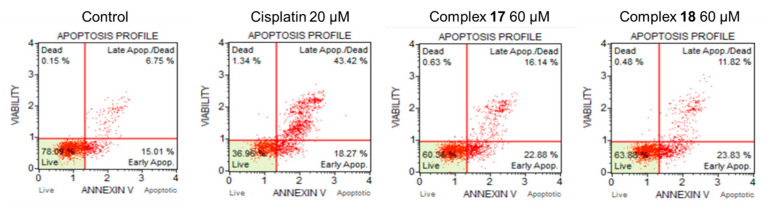
Study of HCT116 cells death mechanism by flow cytometry.

The study of the induction of apoptosis/necrosis for binuclear organometallic complexes Ru(II) showed that complexes **17** and **18** after 72 h of incubation exhibited a lower ability than cisplatin to induce apoptosis of tumor cells (Figure 15).

**Figure 16:**
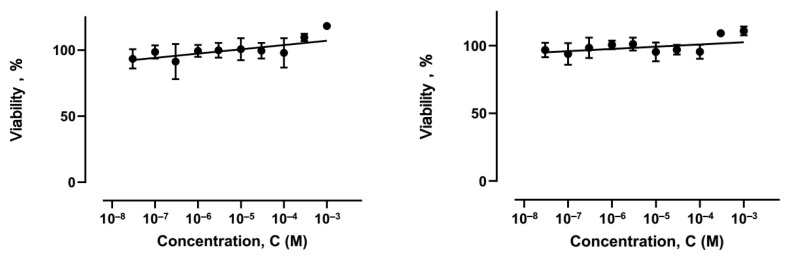
Viability of primary rat dermal fibroblasts during 48 h incubation of complexes **17** (**left**) and **18** (**right**).

The selectivity of the anticancer effect is one of the most important characteristics of a potential anticancer drug. An ideal drug should not harm healthy cells and tissues while still maintaining its ability to fight cancer. To test the effectiveness of two binuclear complexes, **17** and **18**, we examined their impact on the viability and toxicity of rat primary dermal fibroblasts after 48 h of incubation. We used the MTT assay to measure the effects (Figure 16). Both compounds showed no signs of toxicity at concentrations ranging from 0.03 to 1000 micromoles per liter.

The data obtained indicate the prospects of cationic metalacyclic structures with pyridone ligands as a basis for further modification and development of antitumor drugs from various metals, which will ensure the stability of the structure while maintaining the ability of the metal center to bind to biological targets and good solubility in water.

## 3. Materials and Methods

### 3.1. General

All solvents were purified and degassed before use [27]. The NMR spectra were recorded on a Bruker Avance 600 spectrometer (Bruker, Billerica, MA, USA) at 298 K at 600.13 (^1^H) and 150.92 (^13^C{^1^H}) MHz. Two-dimensional NMR measurements were carried out using standard pulse programs. The ^1^H and ^13^C NMR spectra were calibrated by the residual solvent signal, DMSO-d6. Elemental analysis was performed with the MicroCube Elementar analyzer (Elementar, Ronkonkoma, NY, USA). Electrospray ionization (ESI) mass spectra were recorded using a TSQ Endura (Thermo Fisher Scientific, Waltham, MA, USA) instrument. Each analyzed compound was dissolved in methanol (HPLC grade) and injected directly into the ionization source through a syringe pump. The spectra were recorded during 30 s in the *m/z* range 150–1400 in both positive and negative ionization modes with spray voltages of 3.4 and 2.5 kV, respectively. The human HCT116 colorectal carcinoma, A549 non-small cell lung carcinoma, MCF7 breast adenocarcinoma, and SW480 colon adenocarcinoma cell lines were obtained from the European collection of authenticated cell cultures (ECACC; Salisbury, UK).

Single crystals of **4**–**6**, **16**, and **17** were investigated on a Bruker D8 QUEST single-crystal X-ray diffractometer (Bruker) equipped with a PHOTONII detector and microfocus Mo-target X-ray tube (λ = 0.73071 Å). The absorption correction was performed using a multi-scan routine, as implemented in SADABS (Version 2016/2) [28]. Crystal structure solution and refinement were performed using a SHELX-2018/3 package [29]. Atomic positions were located using dual t methods and refined using a combination of Fourier synthesis and least-square refinement in isotropic and anisotropic approximations. All C-H hydrogen atoms were placed in ideal calculated positions and refined as riding atoms with relative isotropic displacement parameters taken as *U*_iso_(H) = 1.5*U*_eq_(C) for methyl H atoms and *U*_iso_(H) = 1.2*U*_eq_(C) otherwise. The hydrogen atoms of the OH groups were located from the Fourier density synthesis. Crystallographic parameters and final residuals for the single-crystal XRD experiments are given in Table S1. A summary of crystallographic data for the single-crystal experiment is available from CCDC, ref. number **2443759**–**2443763**.

### 3.2. Synthesis

- ***N*-1-(3-aminopropyl)imidazole-5-hydroxy-2-methyl-4-pyridone (4)**

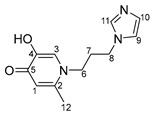


A catalyst, Pd_10%_/C (167 mg, 0.1570 mmol), was added to a solution of compound **1** (1.0 g; 3.1 mmol) in acetic acid (30 mL). The mixture was subjected to hydrogenolysis for 12 h. The palladium catalyst was filtered; acetic acid was evaporated under vacuum. The pure product was isolated by column chromatography on silica gel (eluent—methanol, R_f_ = 0.3). The solvent was evaporated, and the resulting beige hygroscopic powder was dried in vacuum. Yield: 0.4 g (58%, T_decomp._ = 204–206 °C).

^1^H NMR (600.13 MHz, DMSO-d6) δ: 7.65 (s, 1H, H11), 7.36 (s, 1H, H3), 7.23 (s, 1H, H9), 6.91 (s, 1H, H10), 6.05 (s, 1H, H1), 4.01 (t, 2H, J = 7.2 Hz, H8), 3.79 (t, 2H, J = 7.7 Hz, H6), 2.17 (s, 3H, H12), 2.15–2.09 (m, 2H, H7).

^13^C{^1^H} NMR (150.92 MHz, DMSO-d6) δ: 170.5 (C5), 146.8 (C4), 144.4 (C2), 137.3 (C11), 128.6 (C10), 122.3 (C3), 119.2 (C9), 113.6 (C1), 49.5 (C6), 43.2 (C8), 31.1 (C7), 18.1 (C12).

Elemental analysis (C_12_H_15_N_3_O_2_*0.7H_2_O) calculated (%): C 58.62, H 6.72, N 17.09; found (%): C 58.43, H 6.51, N 17.29.

- **N-1-(3-aminopropyl)imidazole-3-hydroxy-2-methyl-4-pyridone (5)**

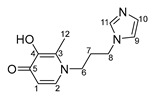


A catalyst, Pd_10%_/C (822 mg, 0.8 mmol), was added to a solution of compound **2** (5.0 g; 15.4 mmol) in acetic acid (100 mL). The mixture was subjected to hydrogenolysis for 12 h. The palladium catalyst was filtered; acetic acid was evaporated under vacuum. The pure product was isolated by column chromatography on silica gel (eluent—methanol, R_f_ = 0.3). The solvent was evaporated, and the resulting beige hygroscopic powder was dried in vacuum. Yield: 2.4 g (67%, T_decom._ = 79–82 °C)

^1^H NMR (600.13 MHz, DMSO-d6) δ: 7.67 (s, 1H, H11), 7.51 (d, 1H, J = 7.3 Hz, H2), 7.23 (s, 1H, H9), 6.92 (s, 1H, H10), 6.11 (d, 1H, J = 7.3 Hz, H1), 4.02 (t, 2H, J = 7.1 Hz, H8), 3.89 (t, 2H, J = 7.7 Hz, H6), 2.21 (s, 3H, H12), 2.15–2.09 (m, 2H, H7).

^13^C{^1^H} NMR (150.92 MHz, DMSO-d6) δ: 168.9 (C5), 145.5 (C4), 137.4 (C2), 137.3 (C11), 128.5 (C10), 128.4 (C3), 119.2 (C9), 110.7 (C1), 50.1 (C6), 43.2 (C8), 31.4 (C7), 11.1 (C12).

Elemental analysis (C_12_H_15_N_3_O_2_*1.0H_2_O) calculated (%): C 57.36, H 6.82, N 16.72; found (%): C 57.19, H 6.32, N 16.79.

- **N-1-(3-aminopropyl)imidazole-3-hydroxy-2-ethyl-4-pyridone (6)**

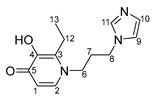


A catalyst, Pd_10%_/C (354 mg, 0.33 mmol), was added to a solution of compound **3** (2.2 g; 6.7 mmol) in acetic acid (60 mL). The mixture was subjected to hydrogenolysis for 12 h. The palladium catalyst was filtered; acetic acid was evaporated under vacuum. The pure product was isolated by column chromatography on silica gel (eluent—methanol, R_f_ = 0.3). The solvent was evaporated, and the resulting beige hygroscopic powder was dried in vacuum. Yield: 1.1 g (69%, T_decomp._ = 122–125 °C)

^1^H NMR (600.13 MHz, DMSO-d6) δ: 7.66 (s, 1H, H11), 7.50 (d, 1H, J = 7.3 Hz, H2), 7.23 (s, 1H, H9), 6.92 (s, 1H, H10), 6.12 (d, 1H, J = 7.3 Hz, H1), 4.04 (t, 2H, J = 7.0 Hz, H8), 3.87 (t, 2H, J = 7.8 Hz, H6), 2.58 (q, 2H, J = 7.5 Hz, H12), 2.15–2.09 (m, 2H, H7), 1.05 (t, 3H, J = 7.5 Hz, H13).

^13^C{^1^H} NMR (150.92 MHz, DMSO-d6) δ: 169.2 (C5), 145.1 (C4), 137.5 (C2), 137.3 (C11), 133.5 (C3), 128.6 (C10), 119.2 (C9), 111.0 (C1), 49.6 (C6), 43.1 (C8), 32.3 (C7), 18.4 (C12), 12.6 (C13).

Elemental analysis (C_13_H_17_N_3_O_2_*0.6H_2_O) calculated (%): C 60.49, H 7.11, N 16.28; found (%): C 60.14, H 7.04, N 16.65.

- **(η6-p-cymene)(N-1-(3-aminopropyl)imidazole-5-hydroxy-2-methyl-4-pyridone)oxalatoruthenium (II) (13)**

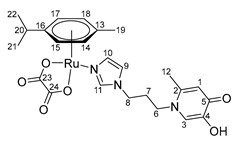


Silver oxalate Ag_2_C_2_O_4_ (130 mg, 0.43 mmol) was added to a solution of ruthenium dimer [(η^6^*-p*-cymene)RuCl_2_]_2_ (131 mg, 0.21 mmol) in 40 mL of H_2_O. The reaction mixture was stirred for 12 h without access to light. The AgCl precipitate was filtered off, and the solvent was evaporated under vacuum. The resulting ruthenium complex was dissolved in 18 mL of MeOH, and a solution of compound **4** (100 mg, 0.43 mmol) in 2 mL of MeOH was added. The reaction mixture was stirred for 8 h, the solvent was evaporated under vacuum, and the product was isolated by column chromatography on silica gel (eluent—methanol). The solvent was evaporated under vacuum, and the resulting orange powder was dried in vacuum. Yield: 120 mg (50%, T_decomp._ = 143–146 °C).

^1^H NMR (600.13 MHz, DMSO-d6) δ: 8.00 (s, 1H, H11), 7.42 (s, 1H, H9), 7.34 (s, 1H, H3), 6.85 (s, 1H, H10), 6.05 (s, 1H, H1), 5.71 (d, 2H, J = 6.0 Hz, H15, H17), 5.49 (d, 2H, J = 5.9 Hz, H14, H18), 4.10 (t, 2H, J = 7.0 Hz, H8), 3.73 (t, 2H, J = 7.7 Hz, H6), 2.73–2.65 (m, 1H, H20), 2.18–2.09 (m, 5H, H7, H12), 2.05 (s, 3H, H19), 1.21 (d, 6H, J = 6.9 Hz, H21, H22).

^13^C{^1^H} NMR (150.92 MHz, DMSO-d6) δ: 170.5 (C5), 164.8 (C23, C24), 146.8 (C4), 144.4 (C2), 139.4 (C11), 129.0 (C10), 122.2 (C3), 120.6 (C9), 113.6 (C1), 99.4 (C16), 96.6 (C13), 82.5 (C15, C17), 79.1 (C14, C18), 49.3 (C6), 44.6 (C8), 30.6 (C7/C20), 30.5 (C7/C20), 22.2 (C21, C22), 18.2 (C12), 17.3 (C19).

Elemental analysis (C_24_H_29_N_3_O_6_Ru*1.0H_2_O) calculated (%): C 50.17, H 5.44, N 7.31; found (%): C 49.81, H 5.72, N 6.97.

ESI-MS: *m/z*: 558 [M + H]^+^, 580 [M + Na]^+^.

- **(η6-p-cymene)(N-1-(3-aminopropyl)imidazole-3-hydroxy-2-methyl-4-pyridone)oxalatoruthenium (II) (14)**

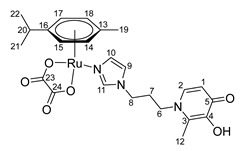


Silver oxalate Ag_2_C_2_O_4_ (130 mg, 0.43 mmol) was added to a solution of ruthenium dimer [(η^6^*-p*-cymene)RuCl_2_]_2_ (131 mg, 0.21 mmol) in 40 mL of H_2_O. The reaction mixture was stirred for 12 h without access to light. The AgCl precipitate was filtered off, and the solvent was evaporated under vacuum. The resulting ruthenium complex was dissolved in 18 mL of MeOH, and a solution of compound **5** (100 mg, 0.43 mmol) in 2.0 mL of MeOH was added. The reaction mixture was stirred for 8 h, the solvent was evaporated under vacuum, and the product was isolated by column chromatography on silica gel (eluent—methanol). The solvent was evaporated under vacuum, and the resulting orange powder was dried in vacuum. Yield: 162 mg (68%, T_decomp._ = 129–132 °C).

^1^H NMR (600.13 MHz, DMSO-d6) δ: 7.99 (s, 1H, H11), 7.48 (d, 1H, J = 7.3 Hz, H2), 7.41 (s, 1H, H9), 6.86 (s, 1H, H10), 6.11 (d, 1H, J = 7.2 Hz, H1), 5.71 (d, 2H, J = 5.9 Hz, H15, H17), 5.49 (d, 2H, J = 5.9 Hz, H14, H18), 4.10 (t, 2H, J = 7.0 Hz, H8), 3.82 (t, 2H, J = 7.5 Hz, H6), 2.74–2.65 (m, 1H, H20), 2.19 (s, 3H, H12), 2.15–2.07 (m, 2H, H7), 2.05 (s, 3H, H19), 1.21 (d, 6H, J = 6.9 Hz, H21, H22).

^13^C{^1^H} NMR (150.92 MHz, DMSO-d6) δ: 168.9 (C5), 164.8 (C23, C24), 145.5 (C4), 139.3 (C11), 137.5 (C2), 129.1 (C10), 128.3 (C3), 120.7 (C9), 110.8 (C1), 99.4 (C16), 96.6 (C13), 82.5 (C15, C17), 79.1 (C14, C18), 49.9 (C6), 44.5 (C8), 30.9 (C7/C20), 30.5 (C7/C20), 22.2 (C21, C22), 17.3 (C19), 11.2 (C12).

Elemental analysis (C_24_H_29_N_3_O_6_Ru*0.8H_2_O) calculated (%): C 50.48, H 5.40, N 7.36; found (%): C 50.19, H 5.65, N 6.88.

ESI-MS: *m/z*: 558 [M + H]^+^, 580 [M + Na]^+^.

- **(η6-p-cymene)(N-1-(3-aminopropyl)imidazole-3-hydroxy-2-ethyl-4-pyridone)oxalatoruthenium (II) (15)**

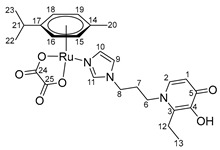


Silver oxalate Ag_2_C_2_O_4_ (126 mg, 0.42 mmol) was added to a solution of ruthenium dimer [(η^6^*-p*-cymene)RuCl_2_]_2_ (128 mg, 0.21 mmol) in 50 mL of H_2_O. The reaction mixture was stirred for 12 h without access to light. The AgCl precipitate was filtered off, and the solvent was evaporated under vacuum. The resulting ruthenium complex was dissolved in 23 mL of MeOH, and a solution of compound **6** (103 mg, 0.42 mmol) in 2 mL of MeOH was added. The reaction mixture was stirred for 8 h, the solvent was evaporated under vacuum, and the product was isolated by column chromatography on silica gel (eluent—methanol). The solvent was evaporated under vacuum, and the resulting orange powder was dried in vacuum. Yield: 131 mg (55%, T_decomp._ = 170–173 °C).

^1^H NMR (600.13 MHz, DMSO-d6) δ: 7.99 (s, 1H, H11), 7.47 (d, 1H, J = 7.3 Hz, H2), 7.42 (s, 1H, H9), 6.86 (s, 1H, H10), 6.12 (d, 1H, J = 7.3 Hz, H1), 5.71 (d, 2H, J = 6.1 Hz, H16, H18), 5.49 (d, 2H, J = 6.1 Hz, H15, H19), 4.12 (t, 2H, J = 7.1 Hz, H8), 3.83 (t, 2H, J = 7.7 Hz, H6), 2.73–2.65 (m, 1H, H21), 2.61 (q, 2H, J = 7.4 Hz, H12), 2.16–2.09 (m, 2H, H7), 2.04 (s, 3H, H20), 1.21 (d, 6H, J = 6.9 Hz, H22, H23), 1.07 (t, 3H, J = 7.4 Hz, H13).

^13^C{^1^H} NMR (150.92 MHz, DMSO-d6) δ: 169.2 (C5), 164.8 (C24, C25), 145.1 (C4), 139.3 (C11), 137.5 (C2), 133.5 (C3), 129.1 (C10), 120.7 (C9), 111.0 (C1), 99.4 (C17), 96.6 (C14), 82.5 (C16, C18), 79.1 (C15, C19), 49.4 (C6), 44.5 (C8), 31.8 (C7), 30.5 (C21), 22.1 (C22, C23), 18.5 (C12), 17.3 (C20), 12.7 (C13).

Elemental analysis (C_25_H_31_N_3_O_6_Ru*1.8H_2_O) calculated (%): C 49.79, H 5.78, N 6.97; found (%): C 49.31, H 5.37, N 7.05.

ESI-MS: *m/z*: 572 [M + H]^+^, 594 [M + Na]^+^.

- **Di-µ-(1-(3-(1H-imidazol-1-yl)propyl)-6-methyl-4-oxo-1,4-dihydropyridin-3-olate)-bis[(η6-p-cymene)ruthenium (II)] chloride (16)**

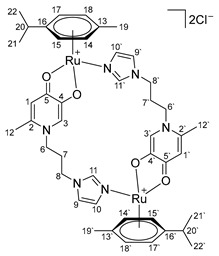


Sodium methoxide (25 mg, 0.45 mmol) was added to a solution of compound **4** (100 mg, 0.43 mmol) in 15 mL of methanol. The reaction mixture was stirred for 3 h, then ruthenium dimer [(η^6^*-p*-cymene)RuCl_2_]_2_ (131 mg, 0.21 mmol) was added and stirred for another 12 h. The solution was filtered, and the solvent was evaporated under vacuum. The resulting residue was dissolved in 1 mL of methanol, and the impurities were precipitated with diethyl ether. The pure product was obtained by crystallization from a mixture of methanol and diethyl ether. The resulting dark orange powder was dried in vacuum. Yield: 181 mg (84%, T_decomp._ = 195–200 °C).

^1^H NMR (600.13 MHz, DMSO-d6) δ: 7.55 (s, 2H, H11, H11’), 7.39 (s, 2H, H9, H9’), 7.13 (s, 2H, H10, H10’), 7.04 (s, 2H, H3, H3’), 6.16 (s, 2H, H1, H1’), 5.77–5.67 (m, 4H, H15, H15’, H17, H17’), 5.52–5.46 (m, 4H, H14, H14’, H18, H18’), 4.20–4.05 (m, 4H, H8, H8’), 3.17–3.09 (m, 2H, H6, H6’), 2.73–2.64 (m, 2H, H20, H20’), 2.09–1.98 (m, 8H, H6, H6’, H12, H12’), 1.88–1.74 (m, 4H, H7, H7’), 1.48 (s, 6H, H19, H19’), 1.26–1.17 (m, 12H, H21, H21’, H22, H22’).

^13^C{^1^H} NMR (150.92 MHz, DMSO-d6) δ: 176.7 (C5, C5’), 160.6 (C4, C4’), 140.3 (C2, C2’), 139.2 (C11, C11’), 131.4 (C10, C10’), 124.1 (C3, C3’), 120.1 (C9, C9’), 110.9 (C1, C1’), 99.3 (C16, C16’), 96.0 (C13, C13’), 81.4 (C15/C15’/C17/C17’), 81.4 (C15/C15’/C17/C17’), 78.8 (C14/C14’/C18/C18’), 78.3 (C14/C14’/C18/C18’), 49.9 (C6, C6’), 44.3 (C8, C8’), 30.6 (C20, C20’), 29.3 (C7, C7’), 22.3 (C21/C21’/C22/C22’), 22.2 (C21/C21’/C22/C22’), 17.6 (C12, C12’), 16.8 (C19, C19’).

Elemental analysis (C_44_H_56_Cl_2_N_6_O_4_Ru_2_*1.0H_2_O*2.0CH_3_OH) calculated (%): C 50.78, H 6.11, N 7.72; found (%): C 50.66, H 6.06, N 7.92.

ESI-MS: *m/z* 538 [M/2 + Cl]^−^, 1043 [M + Cl]^−^, 468 [M − 2Cl]^2+^/[M/2 − Cl]^+^, 971 [M − Cl]^+^.

- **Di-µ-** **(1-(3-(1H-imidazol-1-yl)propyl)-2-methyl-4-oxo-1,4-dihydropyridin-3-olate)** **-bis** **[(η6-p-cymene)ruthenium(II)] chloride (17)**

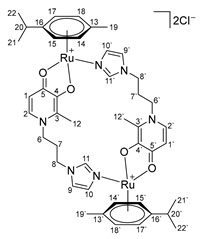


Sodium methoxide (25 mg, 0.45 mmol) was added to a solution of compound **5** (100 mg, 0.43 mmol) in 15 mL of methanol. The reaction mixture was stirred for 3 h, then ruthenium dimer [(η^6^*-p*-cymene)RuCl_2_]_2_ (131 mg, 0.21 mmol) was added and stirred for another 12 h. The solution was filtered, and the solvent was evaporated under vacuum. The resulting residue was dissolved in 1 mL of methanol, and the impurities were precipitated with diethyl ether. The pure product was obtained by crystallization from a mixture of methanol and diethyl ether. The resulting dark orange crystals were dried in vacuum. Yield: 164 mg (76%, T_m.p._ = 150–154 °C).

^1^H NMR (600.13 MHz, DMSO-6d) δ: 7.49 (s, 2H, H11, H11’), 7.34 (s, 2H, H9, H9’), 7.16 (s, 2H, H10, H10’), 6.59 (d, 2H, J = 7.0 Hz, H2, H2’), 6.17 (d, 2H, J = 6.9 Hz, H1, H1’), 5.83–5.68 (m, 4H, H15, H15’, H17, H17’), 5.50 (d, 4H, J = 6.0 Hz, H14, H14’, H18, H18’), 4.28–4.22 (m, 2H, H8, H8’), 4.15–4.02 (m, 2H, H8, H8’), 2.97–2.89 (m, 2H, H6, H6’), 2.76–2.66 (m, 2H, H20, H20’), 2.15–2.04 (m, 12H, H12, H12’, H19, H19’), 2.01–1.93 (m, 2H, H6, H6’), 1.89–1.79 (m, 2H, H7, H7’), 1.74–1.66 (m, 2H, H7, H7’), 1.29–1.14 (m, 12H, H21, H21’, H22, H22’).

^13^C{^1^H} NMR (150.92 MHz, DMSO-d6) δ: 173.9 (C5, C5’), 159.3 (C4, C4’), 138.5 (C11, C11’), 132.3 (C2, C2’), 131.5 (C10, C10’), 120.2 (C9, C9’), 108.9 (C1, C1’), 99.0 (C16, C16’), 96.0 (C13, C13’), 86.3 (C15/C15’/C17/C17’), 85.5 (C15/C15’/C17/C17’), 81.7 (C15/C15’/C17/C17’), 81.3 (C15/C15’/C17/C17’), 78.8 (C14/C14’/C18/C18’), 78.6 (C14/C14’/C18/C18’), 49.9 (C6, C6’), 44.1 (C8, C8’), 30.7 (C20, C20’), 30.0 (C7, C7’), 22.2 (C21, C21’, C22, C22’), 17.6 (C19, C19’), 11.0 (C12, C12’).

Elemental analysis (C_44_H_56_Cl_2_N_6_O_4_Ru_2_*2.0H_2_O*1.0CH_3_OH) calculated (%): C 50.32, H 6.01, N 7.82; found (%): C 50.34, H 5.73, N 7.41.

ESI-MS: *m/z*: 538 [M/2 + Cl]^−^, 1043 [M + Cl]^−^, 468 [M − 2Cl]^2+^/[M/2 − Cl]^+^, 971 [M − Cl]^+^.

- **Di-µ-(1-(3-(1H-imidazol-1-yl)propyl)-2-ethyl-4-oxo-1,4-dihydropyridin-3-olate)-bis[(η6-p-cymene)ruthenium(II)] chloride (18)**

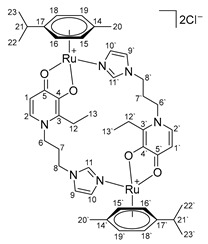


Sodium methoxide (23 mg, 0.42 mmol) was added to a solution of compound **6** (100 mg, 0.4 mmol) in 15 mL of methanol. The reaction mixture was stirred for 3 h, then ruthenium dimer [(η^6^*-p*-cymene)RuCl_2_]_2_ (124 mg, 0.2 mmol) was added and stirred for another 12 h. The solution was filtered, and the solvent was evaporated under vacuum. The resulting residue was dissolved in 1 mL of methanol, and the impurities were precipitated with diethyl ether. The pure product was obtained by crystallization from a mixture of methanol and diethyl ether. The resulting dark orange crystals were dried in vacuum. Yield: 147 mg (70%, T_decomp._ = 160–163 °C).

^1^H NMR (600.13 MHz, DMSO-d6) δ: 7.56 (s, 2H, H11, H11’), 7.33 (s, 2H, H9, H9’), 7.08 (s, 2H, H10, H10’), 6.79–6.73 (m, 2H, H2, H2’), 6.25 (d, 2H, J = 6.9 Hz, H1, H1’), 5.72–5.67 (m, 4H, H16, H16’, H18, H18’), 5.54–5.50 (m, 4H, H15, H15’, H19, H19’), 4.20–4.03 (m, 4H, H8, H8’), 3.16–3.08 (m, 2H, H6, H6’), 2.78–2.60 (m, 4H, H6, H6’, H21, H21’), 2.48–2.38 (m, 4H, H12, H12’), 2.06 (s, 6H, H20, H20’), 1.71–1.58 (m, 4H, H7, H7’), 1.28–1.17 (m, 12H, H22, H22’, H23, H23’), 1.01 (t, 6H, J = 7.4 Hz, H13, H13’).

^13^C{^1^H} NMR (150.92 MHz, DMSO-d6) δ: 174.3 (C5, C5’), 158.9 (C4, C4’), 138.6 (C11, C11’), 137.0 (C3, C3’), 132.2 (C2, C2’), 130.8 (C10, C10’), 120.1 (C9, C9’), 109.1 (C1, C1’), 99.1 (C17, C17’), 96.4 (C14, C14’), 82.0 (C16/C16’/C18/C18’), 81.2 (C16/C16’/C18/C18’), 78.2 (C15/C15’/C19/C19’), 77.9 (C15/C15’/C19/C19’), 50.0 (C6, C6’), 44.1 (C8, C8’), 31.7 (C7, C7’), 30.7 (C21, C21’), 22.2 (C22, C22’, C23, C23’), 18.2 (C12, C12’), 17.6 (C20, C20’), 12.3 (C13, C13’).

Elemental analysis (C_46_H_60_Cl_2_N_6_O_4_Ru_2_*1H_2_O*1.3CH_3_OH) calculated (%): C 51.94, H 6.19, N 7.68; found (%): C 51.49, H 5.74, N 7.48.

ESI-MS: *m/z*: 552 [M/2 + Cl]^−^, 1071 [M + Cl]^−^, 482 [M − 2Cl]^2+^/[M/2 − Cl]^+^, 998 [M − Cl]^+^.

### 3.3. Water Solubility

To determine the solubility of a complex in water using UVI-VIS spectrophotometry, we prepared a series of solutions with different concentrations of the complex. The concentrations were 400 μM, 200 μM, 100 μM, 50 μM, 12.5 μM, and 6.25 μM in water. For each solution, we measured the optical density at 332 nm, which corresponds to the absorption maximum. We then constructed a calibration graph that shows the relationship between the concentration of the complex and the absorption maximum. Next, we prepared a saturated aqueous solution of the complex at 25 °C and diluted it 50 times. We measured the optical density again at 332 nm. We repeated this process three times for each diluted solution. Using the calibration graph, we determined the concentration of each diluted sample. Finally, we calculated the solubility by dividing the concentration by the initial concentration of the saturated solution. All measurements were performed at 25 °C using 96-well plates from Eppendorf, Hamburg (Germany).

### 3.4. Stability

The stability of the Ru(II) complexes was studied by spectrophotometry. For this purpose, absorption spectra were recorded in dry DMF and in a mixture of DMF and water. A 2 mL dry DMF comparison solution with a complex concentration of 100 µM was prepared by diluting 200 µL of the initial solution with a complex concentration of 1 mM in dry DMF and 1.8 mL of dry DMF. To study the stability of the complex for hydrolysis, a working solution was prepared in a mixture of DMF:H_2_O of 9:1; 200 µL of the initial solution with a concentration of 1 mM complex in dry DMF, 1.6 mL of dry DMF, and 200 µL of H_2_O were added. The electronic absorption spectra of the resulting solution were recorded at 25 °C in the range of 270–500 nm with an interval of 30 min.

### 3.5. Cell Death Assays

The antiproliferative activity was studied by MTT assays, as published previously [9].

For the flow cytometry studies, cells were plated into 6-well plates (Eppendorf, Hamburg Germany; HCT-116 cells, 4 × 10^5^ cells in 2 mL of DMEM) and incubated for 24 h. Solutions of complexes in DMSO were prepared immediately prior to the day of the experiments. A cisplatin solution was prepared in DMEM without the addition of DMSO. Cells were treated with either 20 µM of cisplatin, 60 µM of **17**, or 60 µM of **18**. Concentrations corresponded to twofold IC_50_ values based on MTT assays. Cells were incubated for 72 h, collected, washed with PBS, and resuspended in DMEM. Aliquots of cells were processed as recommended in the Muse Annexin V&Dead Cell Kit. Measurements were carried out on a Muse Cell Analyser, Luminex Corp., Austin, TX, USA according to the manufacturer protocol.

### 3.6. Quantum Chemical Calculations (DFT)

A complete optimization of the geometry of all structures was carried out at the level of density functional theory (DFT) using the M06 functional [30] with the help of the Gaussian-09 software package [31]. The basis set 6–31G* was applied to all nonmetal atoms, while the quasi-relativistic Stuttgart pseudopotential describing 28 core electrons (MWB28) and the corresponding contracted basis set [32] augmented by the addition of one f-function (exp 1.111) were used for the Ru atoms. The exponent of this f-function was optimized for an isolated Ru atom in the ground electronic state using the gauopt utility of the Gaussian package. Cartesian basis functions d and f (6d, 10f) were used in all calculations. No symmetry operations were applied to any of the calculated structures. The geometry optimization was performed with consideration to solvent effects, applying the polarizable continuum model in the SMD version [33] with DMSO taken as solvent. The Hessian matrix was calculated analytically for the optimized structures in order to prove the location of the correct minima (no imaginary frequencies) and to estimate the thermodynamic parameters, the latter being calculated at the temperature of 298.15 K and pressure of 1 atm. The experimental X-ray structure of **17** was used as the initial geometry for the optimization of the dimeric complex.

## 4. Conclusions

Thus, new imidazole ligands with a pyridone fragment and ruthenium complexes were obtained. The synthesized ruthenium metallacycles showed good water solubility and antitumor activity and proved stable in aqueous solutions. As well, they showed no toxic effect on healthy cell models. All these data indicate the prospects of such cationic metallacyclic structures with pyridone–imidazole ligands as a basis for further modification and development of various metals antitumor drugs, which, on the one hand, will ensure the water solubility and stability of the structure and, on the other hand, will preserve the ability of the metallocentre to interact with biological targets and exhibit antitumor activity.

## Figures and Tables

**Table 1:** The solubility of binuclear metallacycles **16**–**18** in water.

Compound	Solubility in Water, g/100 mL
**16**	18.4 ± 0.4
**17**	>20
**18**	14.3 ± 0.2

**Table 2:** IC_50_ values of Ru(II) ligands and complexes (72 h incubation).

Compound	IC_50_, μM			
	A549	HCT116	MCF7	SW480
cisplatin	20 ± 2	10 ± 4	15 ± 5	18 ± 5
**4**	>200	>200	>200	nd
**5**	120 ± 13	131 ± 6	153 ± 24	nd
**6**	97 ± 14	91 ± 11	118 ± 18	nd
**13**	>200	>200	>200	nd
**14**	118 ± 15	133 ± 17	124 ± 20	nd
**15**	104 ± 18	114 ± 17	113 ± 20	nd
**16**	>200	>200	>200	>200
**17**	50 ± 8	34 ± 5	45 ± 5	25 ± 4
**18**	28 ± 4	33 ± 5	34 ± 5	26 ± 2

## Data Availability

Data will be made available on request.

## Supplementary Materials

The following supporting information can be downloaded at https://www.mdpi.com/article/10.3390/ijms26115214/s1.
