# Supplementary material for: Antiproliferative Water-Soluble Mono- and Binuclear Ruthenium Complexes with Pyridone–Imidazole Ligands
Source: Int J Mol Sci. 2025 May 29;26(11):5214. doi: 10.3390/ijms26115214 (PMC12155346; doi:10.3390/ijms26115214)
Supplement: Supplementary file 1 [file ijms-26-05214-s001.zip › ijms-3649558-supplementary.pdf]

Supplementary materials: Antiproliferative water-soluble mono- and binuclear ruthenium complexes with pyridone-imidazole ligands

Ilya A. Shutkov, Nikolai A. Melnichuk, Sofya A. Ovakimyan, Dmitrii M. Mazur, Nataliya E. Borisova, Maxim L. Kuznetsov, Ivan A. Godovikov, Konstantin A. Lyssenko, Dmitrii S. Yakovlev, Alexander A. Spasov, Elena R. Milaeva and Alexey A. Nazarov

Table S1. Crystal data, data collection and structure refinement for **4-6**, **16** and **17**.

Compound	**16**	**17**	**4**	**5**	**6**
**CCDC**	**2443763**	**2443764**	**2443760**	**2443759**	**2443761**
Formula	$C_{50}H_{76}Cl_2N_6O_8Ru_2$	$C_{48}H_{76}Cl_2N_6O_{10}Ru_2$	$C_{12}H_{15}N_3O_2$	$C_{12}H_{19}N_3O_4$	$C_{13}H_{17}N_3O_2$
FW	1162.20	1170.18	233.27	269.30	256.30
T, K	107	110	100	100	100
Crystal system	Monoclinic	Triclinic	Monoclinic	Monoclinic	Monoclinic
Space group	$P2_1/n$	P-1	$P2_1/n$	$P2_1/c$	C2/c
Z (Z′)	2(0.5)	1(0.5)	4(1)	4(1)	8(1)
a (Å)	13.6776(9)	8.3791(3)	7.5732(9)	7.0215(5)	13.265(3)
b (Å)	11.6711(8)	12.2284(5)	18.934(2)	24.898(2)	11.640(2)
c (Å)	18.0410(12)	13.8876(5)	7.7640(8)	7.7499(6)	16.591(3)
α (º)	90	70.8100(10)	90	90	90
β (º)	110.324(2)	83.9900(10)	98.226(4)	105.868(3)	93.77(3)
γ (º)	90	74.2000(10)	90	90	90
Volume (Å^3)	2700.6(3)	1292.94(8)	1101.8(2)	1303.21(17)	2556.3(9)
d_{calc}, g·cm^{-3}	1.429	1.503	1.406	1.373	1.332
μ, cm^{-1}	7.14	7.49	0.98	1.04	0.94
F(000)	1208	608	496	576	1096
$2q_{max}$, °	58	58	70	57	58
Refl. collected	24258	19440	15603	12619	12265
Refl. unique (Rint)	7175	6867	4804	3416	3392
Refl. with I > 2σ(I)	5568	5609	3284	2618	2804
Parameters	311	312	214	193	240

R1	0.0554	0.0446	0.0626	0.0537	0.0489
wR2	0.1308	0.1109	0.1657	0.1274	0.1192
GOF	1.025	1.081	1.046	1.040	1.047
Largest difference in peak / hole (e/Å^3)	2.021/-1.120	1.034/-1.151	0.536/-0.273	0.281/-0.297	0.367/-0.236

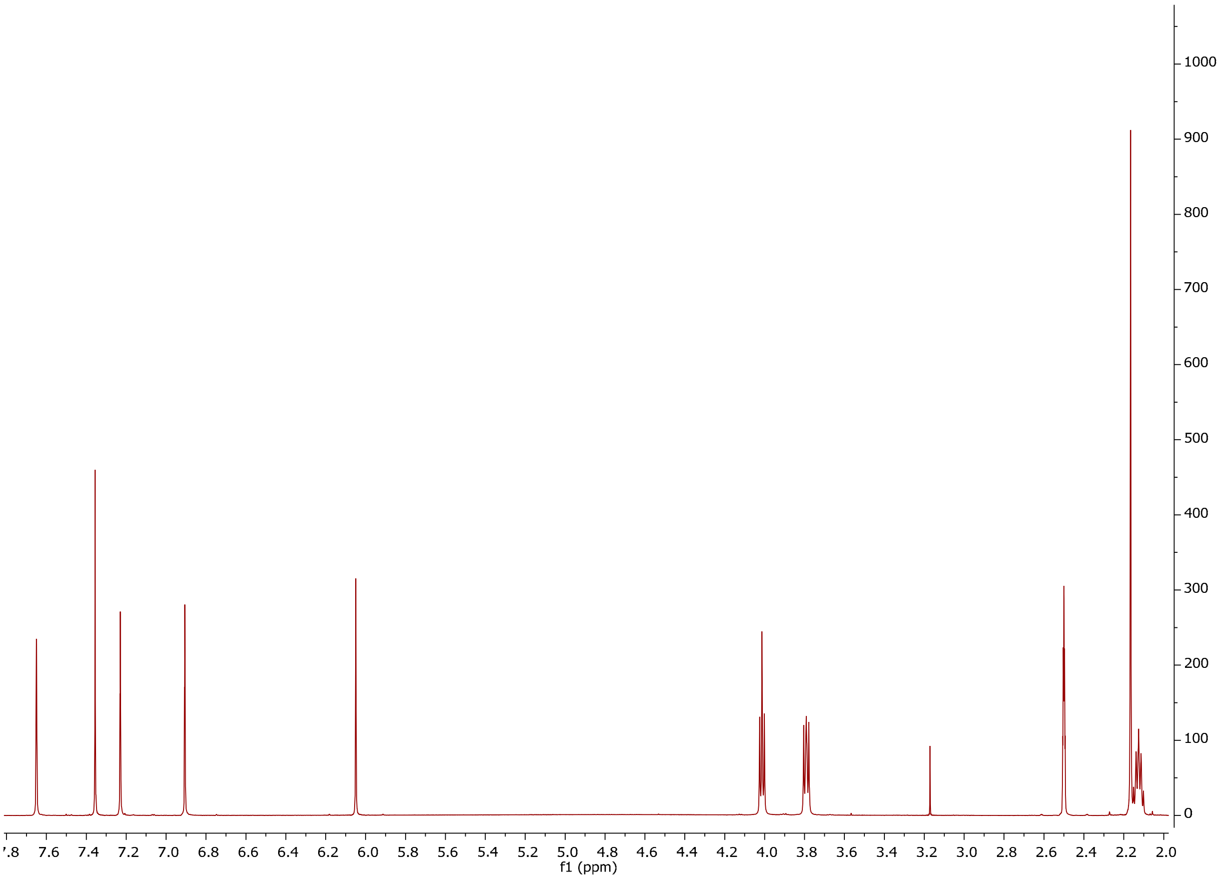

Figure S1. ^{1}H NMR spectrum of ligand **4** in DMSO-d6.

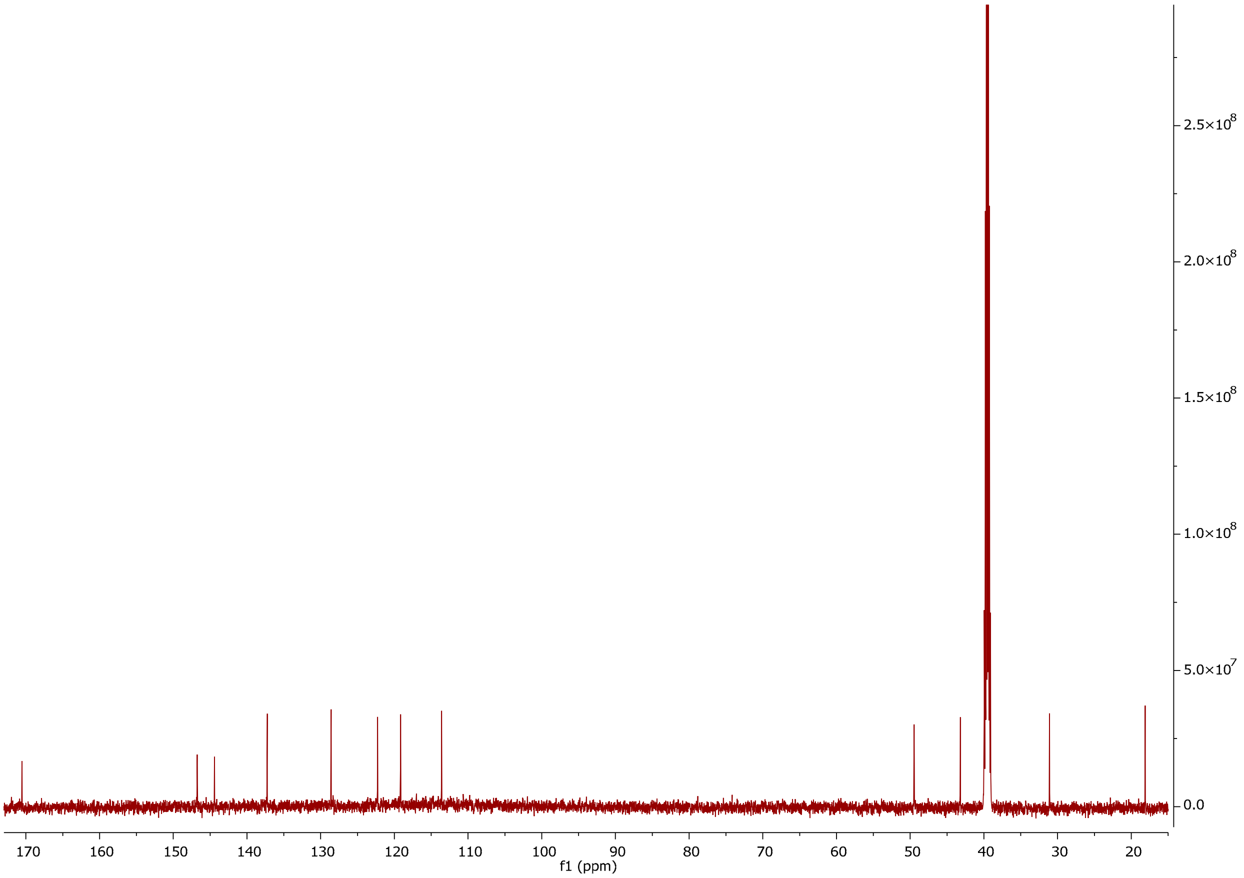

Figure S2. ^{13}C NMR spectrum of ligand **4** in DMSO-d6.

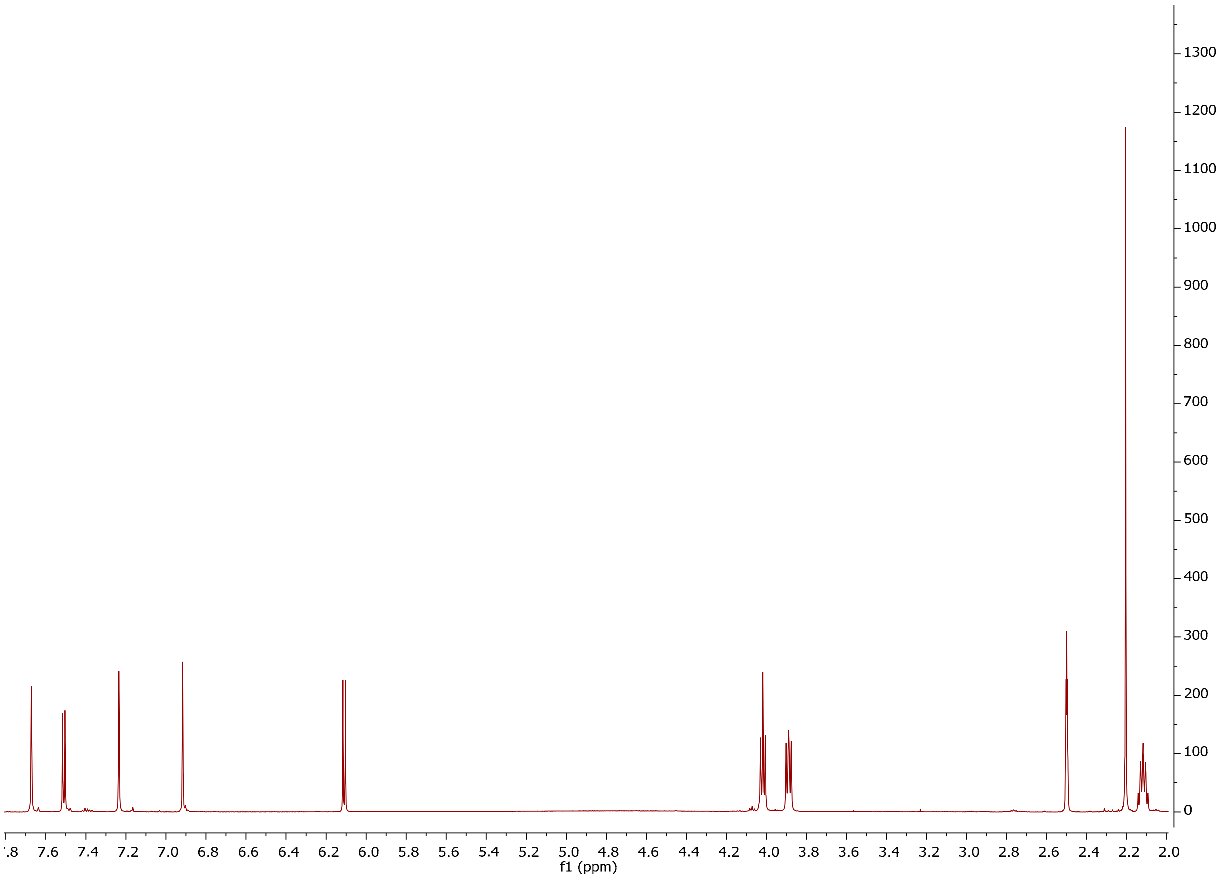

Figure S3. ^{1}H NMR spectrum of ligand **5** in DMSO-d6.

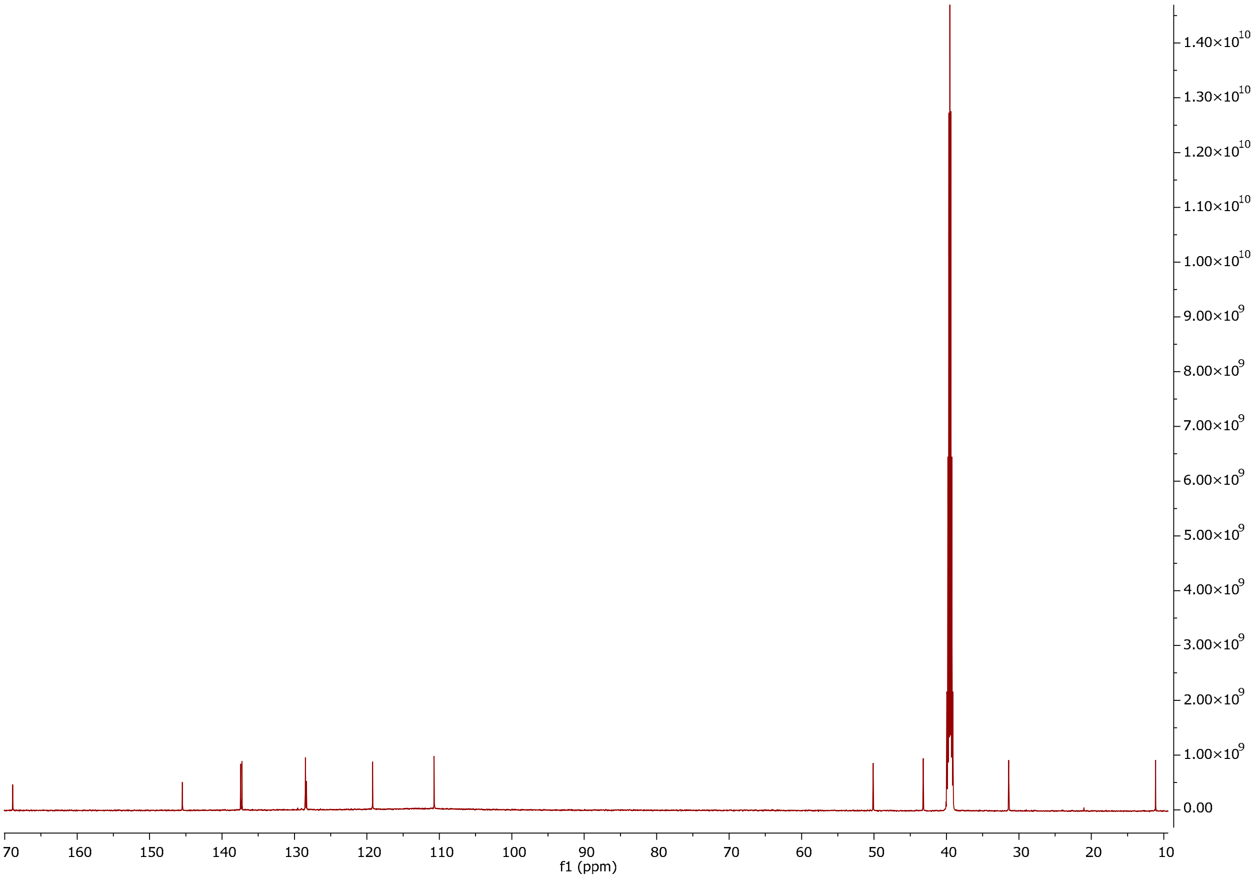

Figure S4. ^{13}C NMR spectrum of ligand **5** in DMSO-d6.

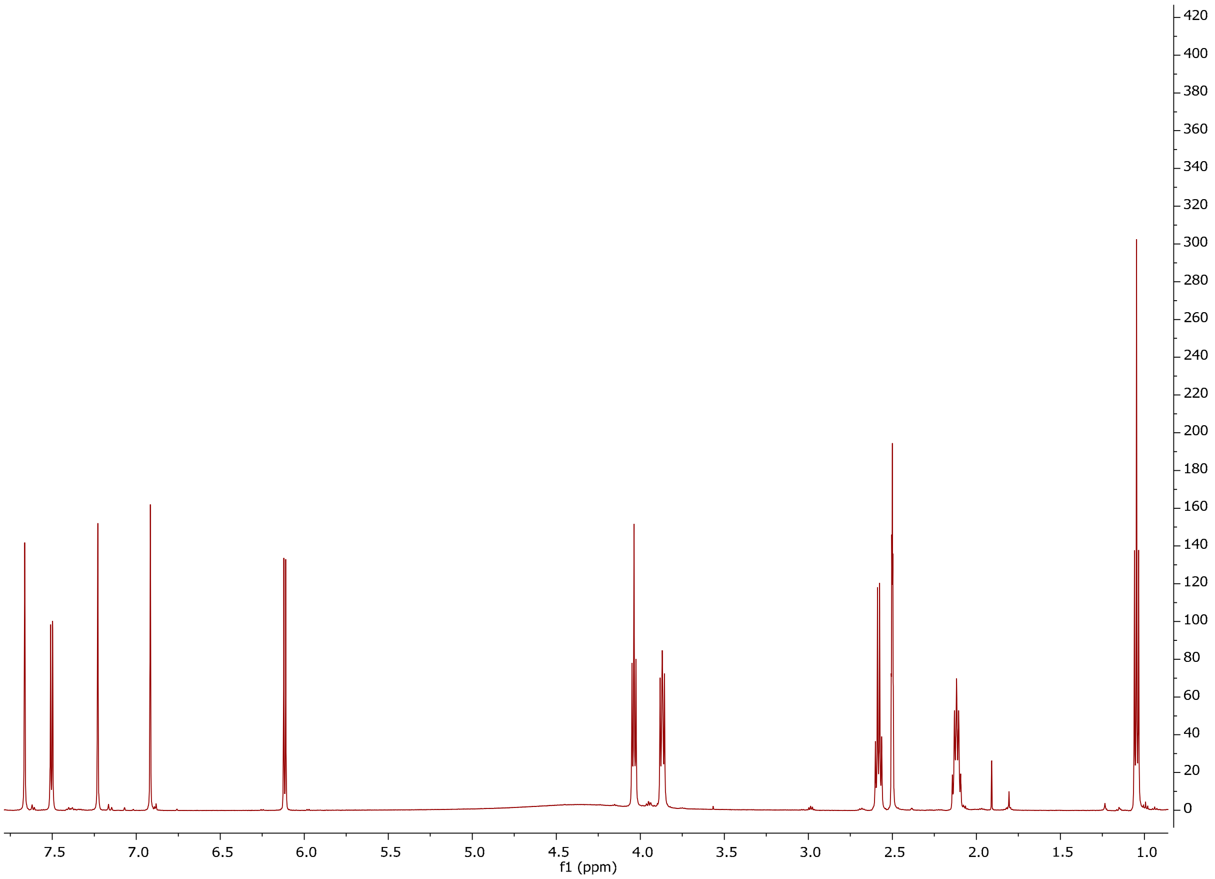

Figure S5. ^{1}H NMR spectrum of ligand **6** in DMSO-d6.

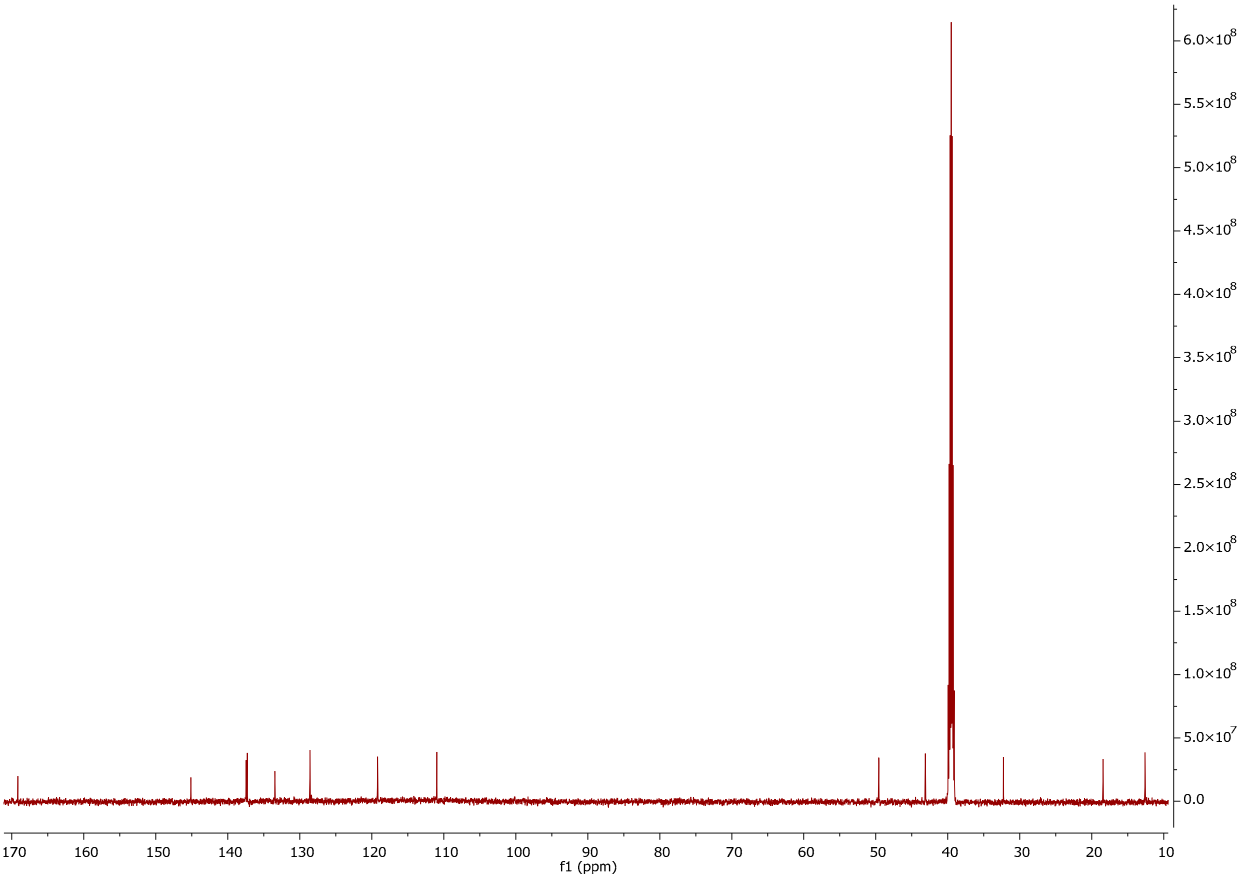

Figure S6. ^{13}C NMR spectrum of ligand **6** in DMSO-d6.

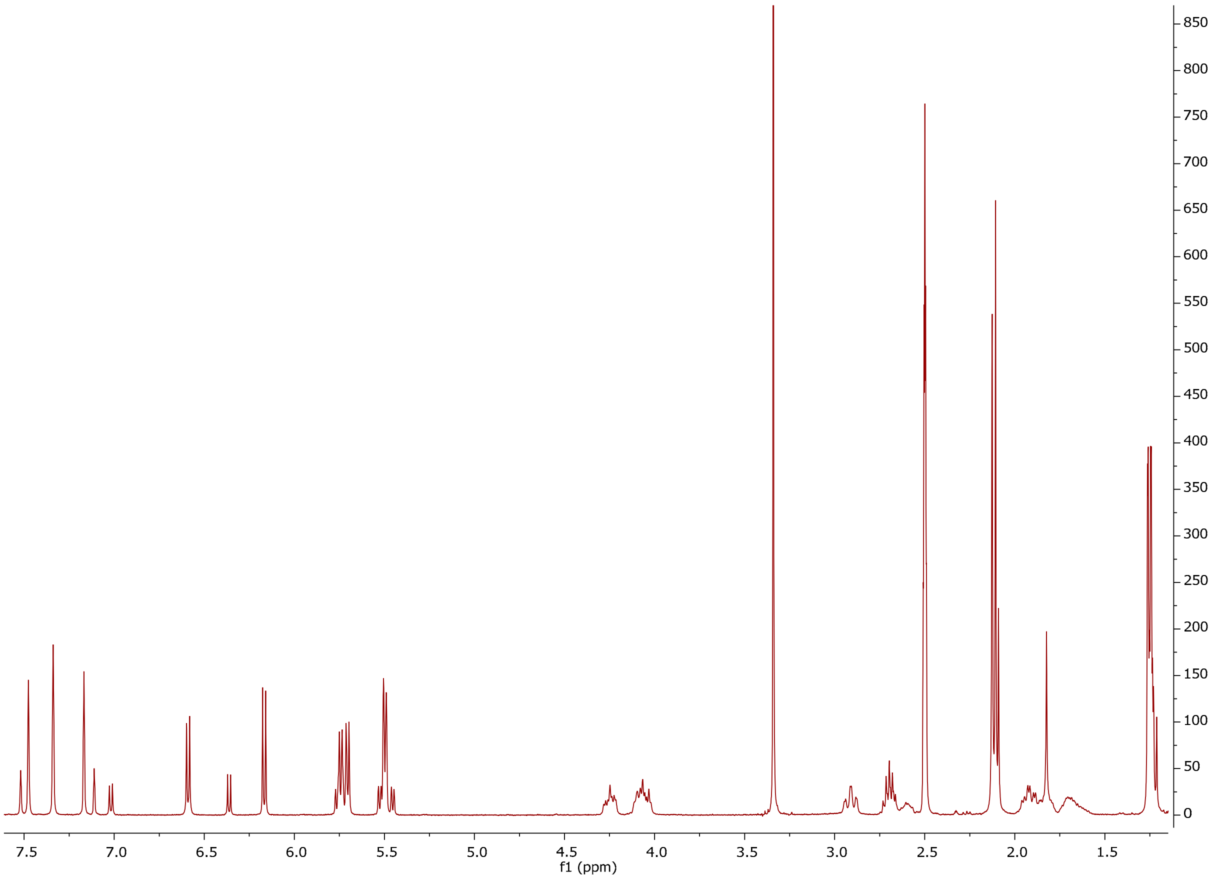

Figure S7. ^{1}H NMR spectrum of complex **17** in DMSO-d6.

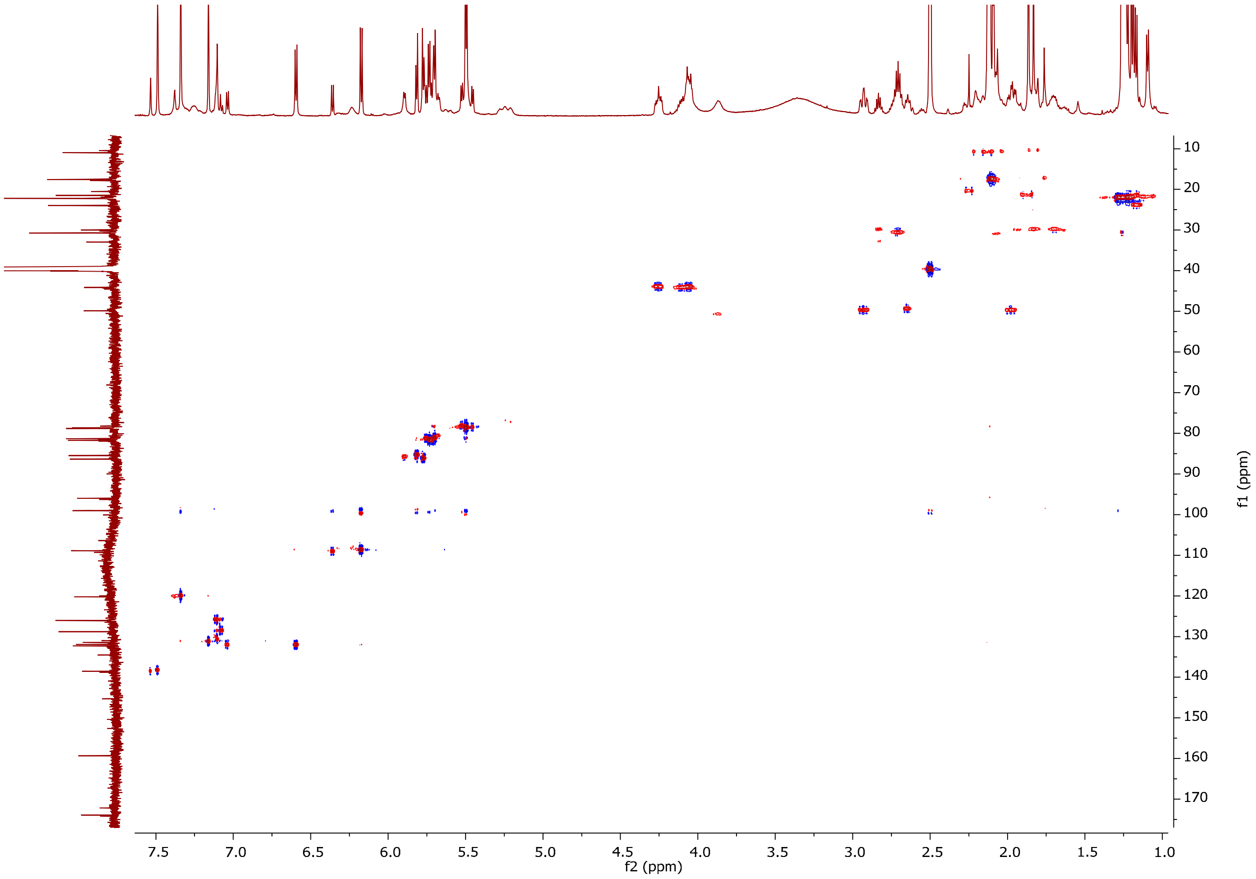

Figure S8. $^{1}H^{13}C$-HSQC spectrum of complex **17** in DMSO-d6.

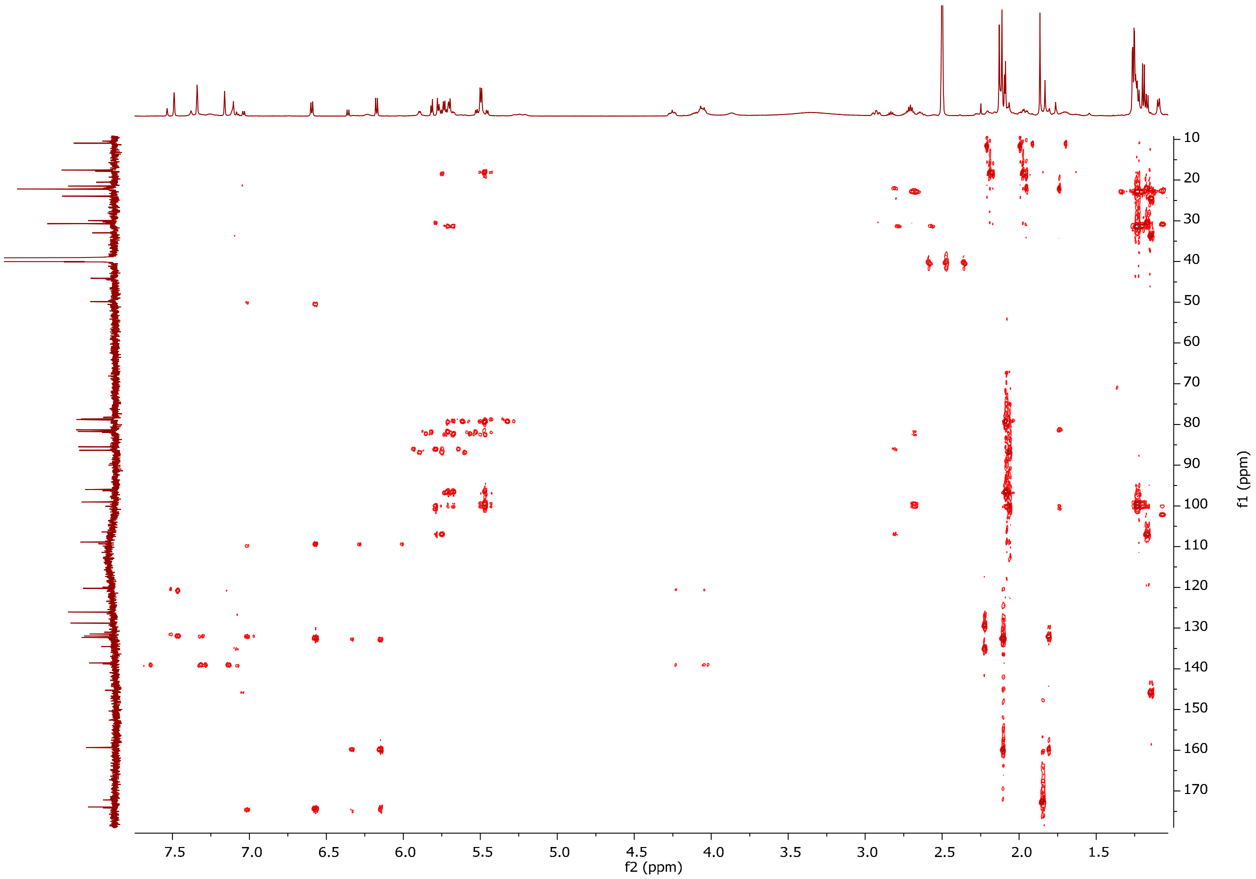

Figure S9. $^{1}H^{13}C$-HMBC spectrum of complex **17** in DMSO-d6.

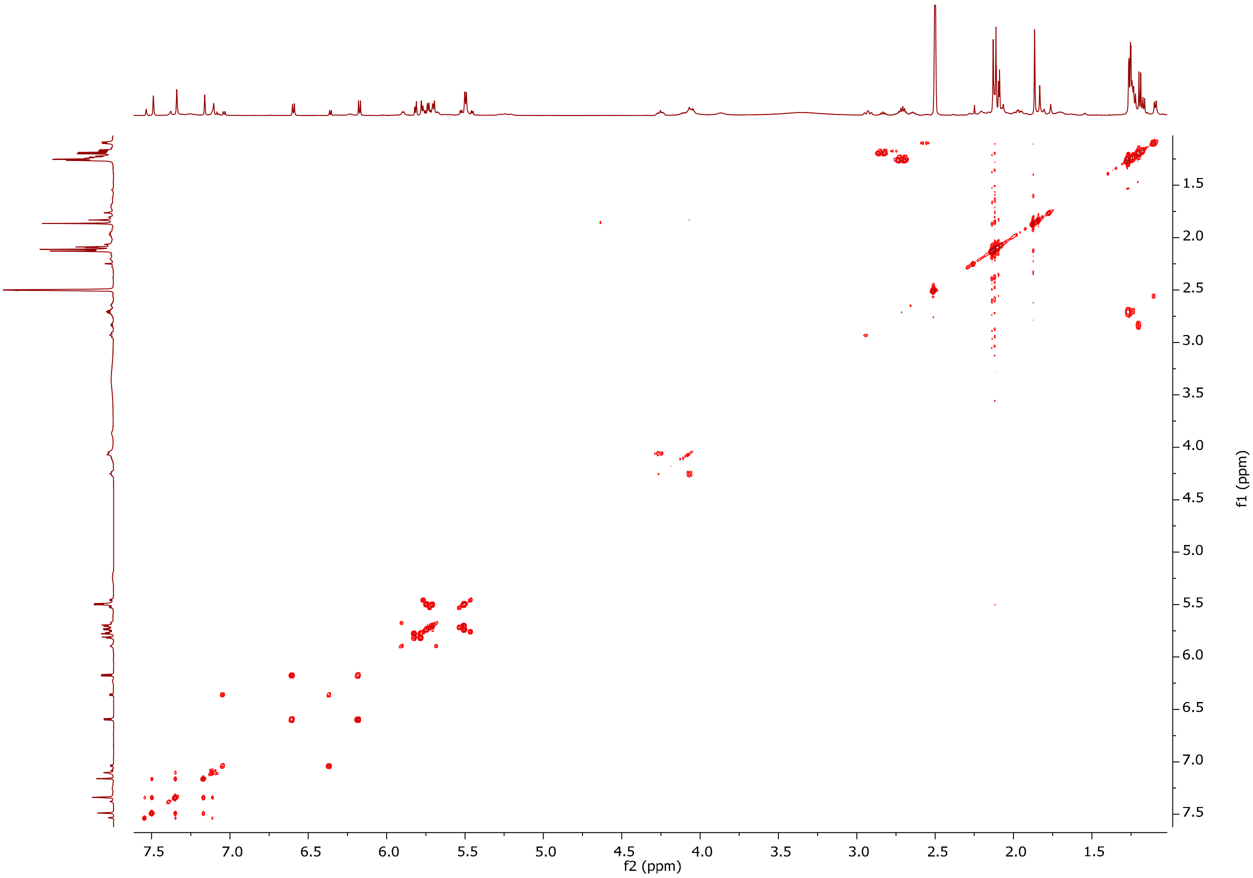

Figure S10. $^1H^1H$-COSY spectrum of complex **17** in DMSO-d6.
